# Soil chemistry and fungal communities are associated with dieback in an Endangered Australian shrub

**DOI:** 10.1007/s11104-022-05724-7

**Published:** 2022-10-01

**Authors:** Samantha E. Andres, Nathan J. Emery, Paul D. Rymer, Jeff R. Powell

**Affiliations:** 1 Hawkesbury Institute for the Environment, Richmond, New South Wales 2753 Australia; 2 The Australian PlantBank, Australian Botanic Garden, Australian Institute of Botanical Science, Mount Annan, New South Wales 2567 Australia

**Keywords:** Soil ecology, Fungi, Biocontrol, Threatened species, Dieback

## Abstract

**Background and aims:**

Field surveys across known populations of the Endangered *Persoonia hirsuta* (Proteaceae) in 2019 suggested the soil environment may be associated with dieback in this species. To explore how characteristics of the soil environment (e.g., pathogens, nutrients, soil microbes) relate to dieback, a soil bioassay (Experiment 1) was conducted using field soils from two dieback effected *P. hirsuta* populations. Additionally, a nitrogen addition experiment (Experiment 2) was conducted to explore how the addition of soil nitrogen impacts dieback.

**Methods:**

The field soils were baited for pathogens, and soil physiochemical and microbial community characteristics were assessed and related to dieback among plants in the field and nursery-grown plants inoculated with the same field soils. Roots from inoculated plants were harvested to confirm the presence of soil pathogens and root-associated endophytes. Using these isolates, a dual culture antagonism assay was performed to examine competition among these microbes and identify candidate pathogens or pathogen antagonists.

**Results:**

Dieback among plants in the field and Experiment 1 was associated with soil physiochemical properties (nitrogen and potassium), and soil microbes were identified as significant indicators of healthy and dieback-affected plants. Plants in Experiment 2 exhibited greater dieback when treated with elevated nitrogen. Additionally, post-harvest culturing identified fungi and other soil pathogens, some of which exhibited antagonistic behavior.

**Conclusion:**

This study identified candidate fungi and soil physiochemical properties associated with observed dieback and dieback resistance in an Endangered shrub and provides groundwork for further exploring what drives dieback and how it can be managed to promote the conservation of wild populations.

**Supplementary Information:**

The online version contains supplementary material available at 10.1007/s11104-022-05724-7.

## Introduction

Globally, there is an increasing occurrence of sudden reductions in the health of native plants resulting in population reductions and localized extinctions (Ciesla et al. 1994). “Dieback” is linked to some of these instances, and involves the partial (e.g., roots, shoots) or entire death of a plant over time. Dieback is of growing concern for the conservation of many native plants and the health of the ecosystems they inhabit (Burgess et al. 2017; Hultberg et al. 2020; Wan et al. 2019). However, the mechanisms driving dieback can be complicated and could result from disease (e.g., soil pathogens or pests) (Grünwald et al. 2012), disturbances (e.g., pollution, development) (Mueller-Dombois 1988; Scarlett et al. 2013), or other abiotic factors (e.g., soil composition, climate warming, drought), many of which often interact in the progression of dieback (Fleischmann et al. 2010; Sangüesa-Barreda et al. 2015; Sapsford et al. 2021). Likewise, dieback in a natural ecosystem may be context dependent. For example, variation in biotic and abiotic components of the soil environment such as soil type (Koepke et al. 2010; McDougall and Liew 2020), soil composition (Crawford and Stone 2015; Halsall et al. 1983; Turner and Lambert 2005), pathogen abundance, or the absence/presence of plant beneficial fungi (Kosawang et al. 2018; Ruiz-Gómez and Miguel-Rojas 2021) might explain differential responses to plant dieback in the landscape. Urgent actions are required to elucidate the drivers of dieback in native plants and to provide sound conservation mitigation solutions.

*Persoonia hirsuta* Pers. is an Endangered shrub native to the Greater Sydney region in New South Wales (NSW), Australia. Significant population decline in recent years and observed dieback by a presently unknown cause has hampered efforts to conserve this species in the landscape and management intervention to address and mitigate the drivers of dieback are urgent. Preliminary field surveys conducted in 2019 (S. Andres, unpublished data) suggested that the soil environment may be an important health determiner for this species. For instance, moderate to severe dieback occurrence was often observed at sites with high abundance of plant species susceptible to the widespread generalist pathogen *Phytophthora cinnamomi*. This pathogen is regarded as one of the world’s 100 worst invasive species (Global Invasive Species Database 2022). Over 250 plant species are known to be susceptible to *P. cinnamomi* in NSW, of which 12% are members of the Proteaceae (McDougall and Liew 2020). Additionally, these preliminary field studies observed higher soil nitrogen beneath plants where severe dieback was observed and no relationship to estimates of disturbance or soil litter composition (S. Andres, unpublished data). Soil nitrogen accumulates in natural ecosystems because of increased agricultural practices (Didham et al. 2015), urban stormwater runoff (Scarlett et al. 2013), or fire exclusion (Jurskis 2005; Turner and Lambert 2005). Past studies have found elevated soil nitrogen increased the onset and severity of dieback in species susceptible to *P. cinnamomi* and other soil pathogens (Jurskis 2005; Scarlett et al. 2013; Sun et al. 2020). However, the effect of nitrogen addition on plant dieback and susceptibility to pathogens in natural ecosystems is still poorly understood (Sun et al. 2020). Furthermore, there are additional biotic (e.g., plant beneficials, pathogen antagonists) and abiotic (e.g., nutrient composition, soil physical properties) characteristics of soils that are also suppressive and conductive to *P. cinnamomi*, which might help or hinder the onset of dieback in a species (Albornoz et al. 2017; Broadbent and Baker 1974; McDougall and Liew 2020; Moreira and Martins, 2005).

For species susceptible to dieback like *P. hirsuta*, it is necessary to investigate characteristics of soils associated with healthy and dieback-affected individuals in the field and to explore the effect of the soil environment on plant dieback. Directly linking observed responses to specific dieback drivers in ecological experiments can be difficult. That is, if the pathosystem is not known, traditional experimental approaches such as susceptibility or manipulation studies may not be feasible as field environments often possess complex disease etiologies (Scarlett et al. 2013; Wan et al. 2019). Bioassays involving plants grown under controlled conditions and inoculated with field soils from healthy and dieback-affected individuals are a useful tool to begin to understand plant-dieback interactions. Within these experiments, soils and roots can be screened to detect the presence and functional importance of pathogens to observed plant dieback (Burgess et al. 2021; Rigg et al. 2018). Additionally, characterizing the composition of microbial communities or abiotic aspects of soils can identify significant soil indicators of healthy or dieback-affected individuals (Broadbent and Baker 1974; Baird et al. 2014; Kosawang et al. 2018; Byers et al. 2020). Such findings have the potential to guide the implementation of microbial inoculum for biocontrol or other soil amendments for use in controlling dieback in the landscape (Byers et al. 2020; Wilkinson et al. 2001).

The present study describes two experiments conducted using field-collected soils from locations of *P. hirsuta* where plants exhibited varying degrees of dieback, to assess potential edaphic drivers of dieback*.* The first experiment- a soil bioassay, compared dieback of *P. hirsuta* individuals in the field to *Pityopsis pinifolia* individuals inoculated with field soils collected beneath eighteen *P. hirsuta* focal plants at two in situ populations. The second experiment aimed to explore differences in dieback under ambient and elevated nitrogen conditions for *P. pinifolia* and *P. hirsuta* individuals growing in two of the soils used in experiment 1. In experiment 1, we assume variation in the soil environment and *P. cinnamomi* or other soil pathogens may be involved in driving dieback in this species. In experiment 2, we expect to observe greater dieback among plants with elevated nitrogen if soil pathogens are present in the soils of inoculated plants.

## Materials and methods

### Field methods (Experiments 1 and 2)

#### Site selection, sampling, and sample preparation

Soils were sampled from beneath nine *P. hirsuta* focal plants each at of two populations (n = 18): Yanderra (34.1835°S, 150.3404°E) and Parr State Conservation Area (33.2230°S, 150.5508°E; referred to as Parr hereafter). These two sites are the largest Southern and Northern *P. hirsuta* populations, respectively. Plants were selected haphazardly to ensure that both healthy and dieback-affected individuals were selected among each population and the initial dieback status of each plant was scored according to an observational scale outlined in Fig. S1. Soil was collected beneath each focal plant at Parr and Yanderra on 8 May 2021 and 10 May 2021, respectively. Beneath each plant, the top litter layer was carefully removed (to ensure only soil was collected) and a total of 3 L of soil was sampled from three locations around the focal plant to a depth of 8 cm with a hand trowel. An additional 6 L of soil was sampled from one focal plant at each population (Parr 5 and Yan 1) for use in the experiment 2. Tools used to collect soil were sterilized with 70% ethanol between each sample. Samples were stored in a cooler and transported to the labs at the Hawkesbury Institute for the Environment (HIE). Soil samples from each focal plant were immediately subsampled into sterile 5 ml tubes and stored at −80 °C for DNA extraction. An additional 400 g of soil was separated from each focal plant for soil baiting (100 g), calculation of plant available water capacity (100 g), and to be sent to the Environmental Analysis Laboratory (EAL) at Southern Cross University to perform analyses on the soil from each block, and source population site (200 g). The remaining bulk soil was used to inoculate the plants in both experiments.

### Laboratory methods on field soil (Experiments 1 and 2)

#### Characterizing the abiotic properties of field soils

The soil parameters analyzed by EAL were: standard Australian testing for exchangeable cations (Ca, Mg, K, Na, H, Al), available phosphorus (Colwell), available nitrogen (Nitrate, Ammonium, Sulfur), pH, effective cation exchange capacity, basic color, basic texture, total nitrogen, total carbon, C:N ratio (LECO Trumac Analyzer), and estimated organic matter (Rayment and Lyons 2011). Soil water holding capacity was determined for each focal plant soil using a gravimetric method (Bilskie and Scientific 2001). For this, a subsample of field soil (~100 g) from each focal plant was placed into a coffee filter, watered thoroughly, and then placed on a tray with an open screen bottom until no more water leaked from the filters. Each focal plant soil was then weighed to get a wet mass (g) and placed into a drying oven at 40 °C for 72 hours. Once dry, the soils were weighed to get the dry mass, and 100 water holding capacity was calculated as:

$$100\%\;water\;capacity=\frac{M\;\mathrm{wet}\;\mathrm{soil}-M\;\mathrm{dry}\;\mathrm{soil}}{M\;\mathrm{dry}\;\mathrm{soil}}$$

Water capacity for each focal plant soil was then used when watering to weight throughout the duration of the experiment.

### Characterization of microbial communities from field soils

#### DNA extraction and sequencing

DNA was extracted from soil subsamples using a Qiagen DNeasy® PowerSoil® kit under the manufacturer’s recommendations. Extracted DNA concentrations were quantified using the NanoDrop 2000 Micro-Volume UV-Vis Spectrophotometer (Thermo Scientific, Wilmington, Delaware, USA). Extracted DNA samples were diluted to 10 ng/uL prior to PCR amplification. DNA samples were sent to the Ramaciotti Centre for Genomics (University of New South Wales, Sydney, NSW, Australia). Amplicons of the V4 region of the bacterial rRNA gene were generated using 0.2 uM 515f (forward primer) and 0.2 uM 806r (reverse primer) (Table 1). Amplicons to identify fungal taxa were generated using 0.2 uM fITS7 (forward primer) and 0.2 uM ITS4 (reverse primer) (Table 1). All amplicons were purified using the Agencourt AMpure XP system (Beckman Coulter, Lane Cove, NSW, Australia) and genomic libraries were prepared using the Nextera XT Index Kit (Illumina, San Diego, CA, USA). Paired-end (2 × 251 bases) sequencing was performed on the Illumina MiSeq platform.

**Table 1:** Primers used for PCR amplification in this study

Name	Direction	Location	Application	Sequence	Reference
DC6	Forward	18S	Oomycetes	GAG GGA CTT TTG GGT AAT CAG AGG GAC TTT TGG GTA ATC A	Cooke et al. (2000)
ITS4	Backward	28S	Oomycetes and fungi	TCC TCC GCT TAT TGA TAT GCT CCT CCG CTT ATT GAT ATG C	Gardes and Burns (1993)
fITS7	Forward	18S	Fungi	GTGARTCATCGAATCTTTG	Ihrmark et al. (2012)
ITS1f	Forward	18S	Fungi	CTT GGT CAT TTA GAG GAA GTA A	Gardes and Bruns (1993)
515f	Forward	16S	Bacteria	5GTGCCAGCMGCCGCGGTAA	Caporaso et al. (2011)
806r	Backward	16S	Bacteria	GGACTACHVGGGTWTCTAAT	Caporaso et al. (2011)

#### Bioinformatics processing

To process the DNA sequencing data, we used the approach described by Bissett et al. (2016) with a few modifications. Contigs were generated from paired-end reads using the ‘fastq_mergepairs’ command in VSEARCH (version v2.3.4; Rognes et al. 2016) using a minimum overlap of 30 (for ITS sequencing) or 200 (for 16S sequencing) base pairs. Initial quality filtering removed DNA sequences containing ambiguous bases and/or homopolymers greater than eight bases in length. Sequences were kept for further analysis if they were within 200–470 (for ITS sequencing) or 251–255 (for 16S sequencing) base pairs in length and contained fewer than 0.5 expected errors. De novo operational taxonomic units (OTUs) at 97% sequence similarity were initially picked using numerically dominant sequences (observed at least two times) using the ‘-cluster_smallmem’ command in VSEARCH. All quality-filtered sequences were mapped at 97% sequence similarity against representative sequences of these OTUs using the ‘-usearch_global’ command in VSEARCH. Non-mapped sequences were subjected to a second round of de novo OTU picking, as above but only using sequences observed at least two times. All initially non-mapped sequences were then mapped against these newly picked OTUs, as above. Non-mapped sequences at this step represent singleton OTUs and were excluded from further analysis.

Putative taxonomic identities for fungal and bacterial OTUs were generated using BLAST (v.2.6.0, Altschul et al. 1990) to compare representative sequences for each OTU to a reference database of gene sequences and taxonomic annotations (bacterial 16S rRNA: greengenes version 13_8_99 (DeSantis et al. 2006); fungal ITS: UNITE version 8.3 (Abarenkov et al. 2021)). Fungal ITS2 sequences were extracted using ITSx (Bengtsson-Palme et al. 2013, v1.1.3) for use during BLAST.

### Isolation of *Phytophthora* species from field soils

#### Soil baiting

Soils from each focal plant (n = 18) in the field were baited using The Center for *Phytophthora* Science and Management (CPSM) standard baiting protocol (Methods can be found in: Burgess et al. 2021). Whole leaves from known *P. cinnamomi* susceptible taxa (*Banksia*, *Camelia*, *Cinnamomum*, *Eucalyptus*, *Lavandula*, *Pinus*, *Persoonia pinifolia* and *Rhododendron)* were collected and placed on top of the water layer for each sample to act as baits. The trays were left at room temperature and baits were monitored for lesions daily for ten days. Soils were rehydrated with Milli-Q water and re-baited a second time once dry. Lesions from baits were cut out with sterile tools (~1 cm^2^) and plated onto the *Phytophthora* selective antibacterial media (17 g of Cornmeal agar (CMA), antibiotics (1 mL Nilstat, 0.1 g Amplimicin, 0.5 mL Rifadin, and 0.025 g Hymexazol (added to the media after autoclaving)), and 1 L of deionized water) (Burgess et al. 2021). Plates were monitored every three days for growth and contamination. Growth resembling *Phytophthora* on any of the initial baited plates were replated under the laminar flow, where the youngest growing colony tissue from a culture was cut out and placed onto a new *Phytophthora* selective media plate. To ensure pure cultures, a third replating onto *Phytophthora* selective media was performed. After the third replating on *Phytophthora* selective media, the youngest colony tissue of observably pure cultures was transferred onto V8 media (17 g of Bacteriological agar 100 mL of V8© vegetable juice, 0.1 g of Calcium Carbonate (CaCO3), 880 mL of water, pH = 7) (Miller 1955). All plates were Parafilm© sealed and stored at room temperature in a Tupperware container with moth balls to prevent mites from contaminating the samples. All soil was autoclaved prior to disposal, and water was mixed with bleach to ensure any pathogens in the soil were killed prior to disposal. All gloves, towels and other small non-living waste was placed into autoclave bags to be sterilised.

#### Identification of baited-isolate cultures

Hyphae from observably pure cultures growing on V8 media were scraped using a sterile scalpel and added to a 2 mL sterile collection tube for DNA extraction using a Qiagen DNeasy® PowerSoil® kit under the manufacture’s recommendations. Following extraction, 20 ul PCR reactions were set up using 4 ul MyTaq® Reaction buffer (Bioline, London UK), *Phythopthora*-specific primers (0.2 uM DC6 (forward primer) and 0.2 uM ITS4 (reverse primer) (Table 1), 0.4 ul MyTaq® DNA Polymerase (Bioline, London UK), and 0.25 ul Bovine Serum Albumin (Thermofisher Scientific, Waltham MA, USA), and 10 ng DNA template and PCR grade water were added to each reaction to reach and final volume of 20 ul. PCR conditions were adapted from Steinrucken et al. (2017), consisting of 96 °C 2 min; 10 cycles of 95 °C for 30 s, 54 °C for 30 s and 72 °C for 1 min; 25 cycles of 95 °C for 30 s, 56 °C for 30 s and 72 °C for 1 min; and a final round of 72 °C for 7 min. Amplification of each isolate was checked using gel electrophoresis on 1% Agarose, with a known *Phytophthora* isolate (*Phytophthora medicaginis*) as a positive control (isolated from chickpea plants grown by Donovin Coles, Western Sydney University). Positive PCR products were further cleaned using Exosap (Thermo Fisher Scientific, Santa Clara) and sequenced at the Sanger sequencing facility at the HIE using 10 ul sequence reactions, set up with 3.2 pmol of ITS4 (sequencing primer), 1–2 ng of purified DNA for every 100 bp, and PCR grade water to bring the volume to 10 ul. Sequence reactions were performed using BigDye Terminator v3.1 cycle sequence kit (Applied Biosystems). Sequencing data was analyzed using Chromus® V6.1.6 (Biomatters Ltd., Auckland, New Zealand) and BLASTn searches were performed using the National Center for Biotechnology Information (NCBI) database with default parameters. Sequences were assigned a putative taxonomic ranking according to the database match with the highest query coverage and percent identity (Table S1). Additionally, sequences from isolates obtained in this study were compared with isolates from closely related species (also sequenced with ITS) obtained from GenBank (http://blast.ncbi.nlm.nih.gov). All were combined and aligned with MUSCLE (Edgar 2004) and phylogenetic trees were produced using Phylogeny. fr (Dereeper et al. 2008) and can be found in Figs. S8-S15.

### Experimental design and setup (Experiments 1 and 2)

#### Seedling propagation

Seedlings of a closely related and easier to propagate congener (*P. pinifolia*) was used for part of the shade house experiments. While the susceptibility of these two individuals to soil pathogens has not been fully tested, we chose to include *P. pinifolia* seedlings in this study to limit the exposure of Endangered *P. hirsuta* plants to potentially harmful pathogens and increase the total sample size of available plants for use in this experiment. These two species share similar distribution, habitat and are closely related (Holmes et al. 2018). These two species also share many functional traits (Emery et al. 2018; Falster et al. 2021) (e.g., leaf thickness, leaf area, life history, reproduction and fire response, height, growth form, and fine root morphology) associated with pathogen susceptibility (Laliberté et al. 2015; Jin et al. 2021; Shearer et al. 2013) further supporting our comparisons.

Sixty-four *P. pinifolia* tube stock seedlings were grown at the Sutherland Shire Council Nursery in Gymea, NSW for use in Experiments 1 and 2. To generate these seedlings, *P. pinifolia* seeds were collected in November 2019, placed in a plastic bag with damp potting mix, and stored under the benches in the glasshouse for one year. The hard seed coat was removed with sandpaper and seeds were sown in a 50/50 perlite vermiculite mix. Seeds were germinated in April 2020, and seedlings were later transferred to tube stocks in 50/50 perlite vermiculite mix. Seedlings were kept outside for 11 months prior to being transported to the shade house. Additionally, twenty *P. hirsuta* seedlings were grown at the Australian Botanic Garden Mount Annan (ABGMA) for use in experiment 2. Fruits of *P. hirsuta* were collected from an extant population in Yengo National Park in November 2017. Fruits were cleaned and stored at −20 °C in the NSW Seedbank before being germinated in January 2020. Germinants were potted into 50 mm tubes containing a soil mix of crushed quartz and coir fiber with a low organic matter content and placed in a temperature-controlled glasshouse. After three months the plants were re-potted in 90 mm forestry tubes and transferred to a shade house for an additional nine months with bottom-up watering.

#### Experimental design

Both experiments were conducted in the S33 polytunnel at the HIE. For the experiment 1, three *P. pinifolia* plants were randomly assigned to each of the eighteen focal plant soils (n = 54) (Fig. S2a). For experiment 2, four *P. pinifolia* plants and five *P. hirsuta* plants were randomly assigned to one focal plant soil from each population (Parr plot 5 and Yanderra plot 1) and treatment (elevated N, ambient N) (n = 36) (Fig. S2b). *Persoonia pinifolia* replicates from the bioassay for the two focal plant soils in experiment 2 were shared between the two experiments under the ambient N treatment group. On a weekly basis, 100 mL of a 100 mg L^−1^ solution of ammonium nitrate was added to N+ treatment plants in experiment 2 (Scarlett et al. 2013). Plants in both experiments were inoculated with soils from each focal plant on March 19th, 2021. Each plant was carefully removed from its pot and placed into the center of a 140 mm pot. Soil collected from under the relevant focal plant was then placed around the plant. Plants were placed in random rows clustered by soil origin 30 cm apart to reduce the possibility for cross contamination of oomycetes between soil origins (Fig. S3a-b). Plants were watered-to-weight to 100% field capacity just after potting and then twice weekly using the soil water capacity data for each focal plant in beforementioned methods, except during a period of limited campus access because of a COVID-19 outbreak in NSW (July–September 2021) when plant watering was reduced to once weekly.

#### Experimental monitoring and maintenance of experimental seedlings

Monitoring was conducted among all plants in both experiments roughly every three weeks except during a period of limited facility access because of a COVID-19 lockdown (July–September 2021) where plant monitoring was conducted for the second to last (seven weeks later) and final (four weeks later) monitoring events. At each monitoring event, observable measures of above-ground plant height (mm from base of the stem), number of branches (number of end points from any stem), stem defoliation (total mm of defoliation on any part of the plant), stem death (total mm of dead stem on any part of the plant), and dieback (estimated using ocular (Fig. S1) and repeat photography methods) occurred. Photos were taken of each experimental replicate, one photo for each *P. pinifolia* replicate and two for each *P. hirsuta* replicate. Two photos were taken from each *P. hirsuta* replicate because the individuals were larger and more multi-dimensional than the *P. pinifolia* tube stocks. Photos were taken mid-day inside the polytunnel using a white posterboard and a 12MP Nikon Coolpix S3000 digital camera (Fig. S3c). Photos were taken to include the maximum extent of the plant in the field of view while excluding the pot as the pot will interfere with the downstream analysis of the photos (Fig. S3d). Pixels were extracted from photos at each monitoring timepoint in R (R Core Team 2021) using the ‘countColorsInDirectory’ function from the countcolors package (Hooper et al. 2020). Healthy (green) pixels were centered using a RGB triplet of 0.25, 0.45, 0.25 and a radius of 0.2. Dieback pixels were centered using RGB triplets for dead (black) plant material (0.2, 0.2, 0.1), and discoloration (yellow) plant material (0.23, 0.25, 0) with a radius of 0.02 and 0.03 respectively (Fig. S4 for example). Using these values, the proportion of dieback relative to healthy pixels extracted from each photo were used in subsequent analyses.

#### Experimental harvest

**Fig. 1:**
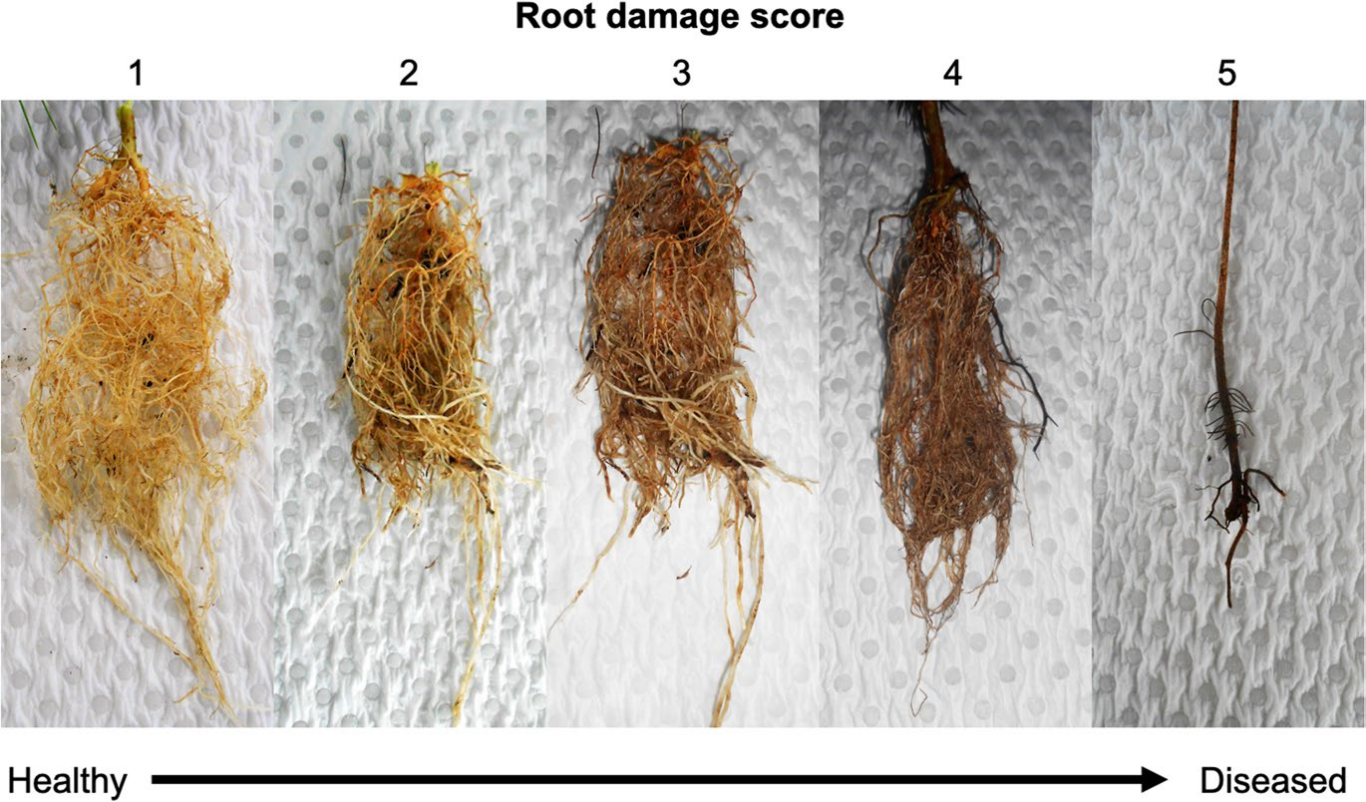
Examples of root damage for *Persoonia* seedlings 187 days post-inoculation with soils from both field sites. Ocular estimation of root system lesions was rated on a scale of 1–5 (1 = no damage, 4 = severe root damage)

All plants were harvested on September 30th, 2021 (187 days post-inoculation). Plants were carefully removed from the 140 mm pots and the roots were washed of remaining soil with water. Photos of the root system were taken for each plant to later characterize dieback using a visual rating system on a scale of 1–5 (1 = no damage, 5 = severe root damage) (Fig. 1). Plants were separated into above and below-ground growth by cutting at the base of the meristem, and fresh root and shoot weights were taken. Ten small root fragments were harvested for all plants in experiments 1 and 2 to evaluate the presence of possible soil pathogens inside plant roots. Ten additional small root fragments were harvested from twelve plants from experiment 1 growing in the four sources of soil inoculum that had a high abundance of soil identified as significant indicators of healthy plants (see methods and results). Using these root fragments, we explored whether these significant indicator taxa could also be found in association with plant roots. A final wet root weight was taken for each plant to account for the subsampled root biomass, then roots and shoots were placed into a dehydrating oven at 60 °C and reweighed after three days.

### Laboratory methods on harvested plants (Experiments 1 and 2)

#### Isolation and identification of root-associated fungi

All root fragments were washed in a 0.5% bleach solution for 30 s and dried with paper towel prior to plating. Ten root fragments from each of the 89 harvested plants were plated onto *Phytophthora* selective media (as described above) in an attempt to reisolate *P. cinnamomi* from plant roots. Plates were monitored for one week for growth and the proportion of plated roots with colonization on *Phytophthora* selective media was recorded for each plant as a variable to be used in later analyses. Plated roots with observable growth were replated two more times on the selective media to obtain pure cultures. Pure cultures were transferred to V8 media prior to DNA extraction, PCR and sequencing using DC6/ITS4 primers and methods described above. The root fragments harvested from twelve plants growing in soils with high fungal indicator taxa abundance from experiment 1 (see below) were plated onto Malt Extract agar with 670 mg/L Rose Bengal, a media that has been found to suppress bacterial growth and successfully isolate fungi and actinomycetes from natural sources (Ottow and Glathe 1968) DNA extraction was conducted using methods described above. To identify these specific isolates, 20 ul PCR reactions were set up using 4 ul MyTaq® Reaction buffer (Bioline, London UK), ITS1f (0.2 uM DC6 (forward primer) and 0.2 uM ITS4 (reverse primer) (Table 1), 0.4 ul MyTaq® DNA Polymerase (Bioline, London UK), and 0.25 ul Bovine Serum Albumin (Thermofisher Scientific, Waltham MA, USA), and 10 ng DNA template and PCR grade water were added to each reaction to reach and final volume of 20 ul. PCR conditions were adapted from Steinrucken et al. (2017), consisting of 94 °C for 5 min; followed by 35 cycles of 94 °C for 30s, 55 °C for 30s and 72 °C for 30s; and a final extension of 72 °C for 5 min (Anderson et al. 2003). All PCR products were checked using gel electrophoresis and Sanger sequencing and taxonomic annotation of all DNA sequences was performed as described above.

#### Dual culture antagonism assay

The antagonism of root-associated fungal isolates against probable pathogen cultures was investigated using a dual culture antagonism assay in which the simultaneous growth of two cultures in vitro were investigated over time. Six isolates that were frequently isolated from plant roots (*Aspergillus* sp.*, Penicillium* sp.*, Trichoderma* sp.*, Fusarium* sp.*, Ilyonectrica* sp.*, and Cylindrocladiella* sp.) and a *P. cinnamomi* culture that was baited from soil were chosen, equating to a total of seven isolates (Table S1). Dual culture assays were set up to explore paired comparisons between each of the seven isolates equating to a total of 21 different paired interactions with five replicates per interaction. For each isolate, a small (~2 cm^2^) sample of the youngest growing colony tissue growing on 1.5% Malt Extract Agar (MEA) was plated onto a new MEA plate approximately 2 cm from the perimeter of the plate (measured with a ruler). Additionally, a separate plate was made for each isolate to serve as a solitary control. Plates were monitored every three days for nine days for quantitative (competitive radial growth) and qualitative (observable in vitro activity suggesting competitive attributes) inhibition.

Quantitative inhibition was evaluated using methods adapted from Rigerte et al. (2019) in which the shared (α) and orthogonal (β) axes of growth were measured to infer direct and indirect measures of competitive spread. These measurements were adapted to include any growth along the shared and orthogonal axes of growth when measuring α and β as some fungi (e.g., *Penicillium*) grew non-radially and instead competitively producing multiple colonies along either axis of growth. The ratio of α/β (the spherical index) was then used to determine the degree of antagonism among the two isolates. Using these methods, quantitative inhibition among pairs was defined when the shared axis of growth for one isolate is greater than its growth orthogonal to the shared axis (α/β > 1 = the antagonist isolate) and the growth of the other isolate along the shared axis is less than its growth orthogonal to the shared axis (α/β < 1 = the inhibited isolate) (see Fig. S5 for more information). Qualitative inhibition was evaluated among isolates using methods outlined in Mejía et al. (2008) in which three forms of observed inhibition were recorded (1) Antibiosis: growth-inhibition determined by the presence of an inhibition zone; (2) competition for substrate: overgrowth of one organism by another; and (3) mycoparasitism: direct parasitism on the hyphae of the pathogen.

### Statistical analyses

All statistical analyses were conducted in the R environment version 4.0.3 (R Core Team 2021). The ‘stats’, ‘car’, ‘lme4’, ‘vegan’ and ‘Hmisc’ packages (Bates et al. 2018; Harrell and Dupont, 2021; Oksanen et al. 2020; R Core Team, 2021) were used to conduct most analyses. All linear mixed effects models were fit with the function ‘lmer’ (with population inoculum included as a random effect), and linear fixed effects models were fit with the function ‘lm’, all ANOVA statistics were obtained with the function ‘Anova (…, type = ‘II’, test = ‘F’)’, and t-tests were fit with the function ‘t.test’ (R Core Team 2021). Pairwise comparisons and significant interaction effects were explored using the functions ‘emmeans’ or ‘emtrends’ (Lenth et al. 2018).

#### Calculation of growth rates and dieback response

To explore the overall effect of soil inoculum on plant growth and dieback status, average growth and dieback rates for measured variables (plant height, ocular dieback, proportion of dieback pixels relative to healthy pixels) were calculated by extracting the slope of a linear model over days since initial monitoring for each plant. Measurements of the stem defoliation, and stem death were subsetted to include the final monitoring timepoint (187 days post inoculation) and converted to a proportion based on the total height of each plant. To develop a “dieback response” for each plant, a principal components analysis (PCA) of all summarized dieback measurements (ocular rate, pixel rate, and proportion of defoliation and dead branch material at final timepoint) was performed using the ‘prcomp’ function from the ‘vegan’ library (Dixon 2003). The PC1 axis from this ordination was extracted using the scores function in ‘vegan’ and used as the dieback response. To assist in downstream applications analyzing microbial community data (variation partitioning and dieback grouping for indicator species analysis), we further categorized this dieback response among inoculated plants into two groups using this axis (“low-none” and “moderate-severe”) based on points that were < or > 0 respectively.

#### Estimation of field soil effects on, and relationships among, *P. pinifolia* bioassay responses (Experiment 1)

To confirm that soil inoculum from sites with higher rates of dieback in the field were associated with higher rates of dieback for inoculated seedlings, mixed models using observed dieback among plants in the field, and the dieback response among experimental plants 187 days post-inoculation were fitted. To explore the significance of ecologically relevant edaphic variables (total nitrogen, total carbon, phosphorus, calcium, magnesium, pH, plant available water capacity) in accounting for variation in dieback among field and inoculated plants a multivariate constrained analysis of principle components (CAP) was fitted on standardized edaphic variables with a stepwise model building approach and selection in bi-directional model selection using the ‘ordistep’ function. Mixed models were then fitted using these soil characteristics (potassium and total nitrogen) and the detection of *P. cinnamomi* from baited soils as predictors of dieback among field plants and overall inoculated plant dieback response 187 days post inoculation. We encountered difficulties estimating residual degrees of freedom using the mixed model, and population inoculum explained minimal variation overall, therefore a standard fixed effects model was used for these analyses.

Variation partitioning was used to understand the role of edaphic variables (total nitrogen, total carbon, phosphorus, calcium, magnesium, pH, plant available water capacity), spatial location, and dieback (in the field and among inoculated plants) in explaining differences in bacterial and fungal community datasets. Variation partitioning revealed that inoculated plant dieback response significantly predicted the fungal community composition while no dieback variables explained variation in the bacterial community composition (see results and Fig. S6). Therefore, subsequent analyses on field soils were to further explore variation in the fungal community with an emphasis on the observed binary dieback response among plants inoculated with field soil.

To determine whether specific fungal OTUs were significantly associated with field soils where inoculated plants were exhibiting dieback symptoms, an indicator species analysis was conducted using the ‘multipatt’ function from the ‘indicspecies’ package (De Cáceres and Legendre 2009) with the binary dieback response variable to separate plants into healthy and dieback-affected groups. Indicator species analyses were conducted using all fungal community data, as well as subsets for populations individually (Yanderra and Parr). Significant indicators (*P* < 0.05) with a positive predictive value and sensitivity value greater than 0.75 were included. For OTUs identified as significant indicators and that had been assigned taxonomic labels at the species or genus levels, the FUNGuild database (Nguyen et al. 2016) and additional literature searches were used to determine whether their potential involvement might be via driving (pathogen) or alleviating (pathogen antagonist) dieback.

To explore whether observed dieback above ground was associated with below-ground root damage among inoculated plants experiment 1, linear mixed models were fitted for dieback response, with root damage included as a fixed effect. To explore whether the proportion of roots with colonization on *Phytophthora* selective media also varied for these plants among different levels of root damage, mixed effects models were also fitted. To explore whether above ground-dieback among inoculated plants was associated with the proportion of roots with colonization on *Phytophthora* selective media or root biomass mixed effects models were also fitted. Quantitative inhibition rates (α/β) between microbial cultures in the dual culture assay were first filtered to ensure that α/β > 1 for one isolate and α/β < 1 for the other, and comparisons were made using t-tests for each interaction of isolates.

#### Estimation of nitrogen effects on *P. hirsuta* and *P. pinifolia* dieback responses (Experiment 2)

Measurements of stem defoliation and stem death were converted to a proportion based on the total height of each plant. Linear mixed effects models were fitted for each inoculated plant dieback response variable (ocular dieback, proportion of stem defoliation relative to total plant height, proportion of stem death relative to total plant height, proportion of dieback pixels relative to healthy pixels), and plant height with time, treatment, and species as fixed effects.

Mixed effect models were fitted to explore whether above ground dieback was associated with below-ground root damage and nitrogen addition among inoculated plants in experiment 2. Mixed effects models were also fitted to explore whether the proportion of roots with colonization on *Phytophthora* selective media also varied among different levels of root damage or nitrogen addition. To explore whether above ground-dieback among inoculated plants was associated with the proportion of roots with colonization on *Phytophthora* selective media or root biomass, mixed effects models were also fitted with nitrogen addition treatment included as an additional fixed effect.

## Results

### Field site and soil descriptions

Soils from the field were marginally acidic with a pH from 4.12–4.75, low nitrogen (0–0.2%), and phosphorus (0.11–0.20 mg/kg), and plant available water capacity ranged from 17 to 48%. Soil characteristics varied between both populations with soils from Yanderra characterized by loamy sand with higher nitrogen, carbon, potassium and phosphorus than Parr, while soils from Parr were predominantly characterized by sand with higher soil calcium, and magnesium than Yanderra (Table S2). Dieback in the field ranged from very low or none (0–10%) to moderate (30–40%); of those affected by dieback, symptoms such as leaf discoloration, and/or defoliation were frequently observed (Fig. S1). All isolates detected from the initial soil pathogen baiting process belonged to the genus *Phytophthora* (n = 9). Seven isolates from both populations frequently matched with *P. cinnamomi* in our BLAST searches and clustered with *P. cinnamomi* in our phylogenetic tree (Fig. S8). The other two *Phytophthora* isolates from Parr did not have adequate matches to identify them to species (Table S1).

### Experiment 1 (Bioassay)

#### Comparison of edaphic characteristics and dieback among inoculated plants to plants in the field

**Fig. 2:**
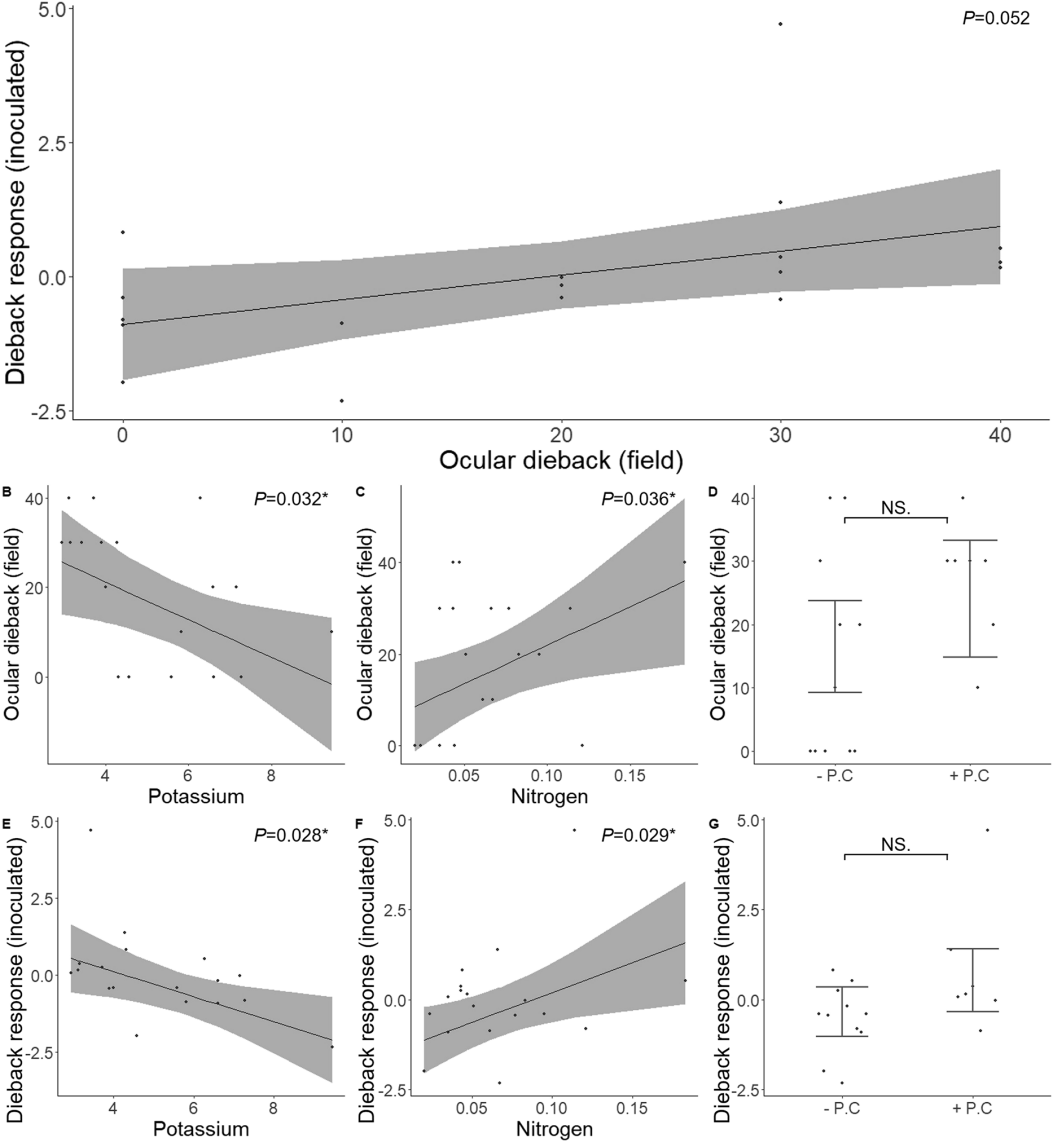
Comparisons of dieback and soil parameters among plants in the field and average dieback response among plants inoculated with field soils. **a**) Relationship between ocular dieback in the field to dieback response among plants inoculated with field soil 187 days post-inoculation, **b**) relationship between ocular dieback in the field and soil potassium, **c**) relationship between average dieback response among inoculated plants and soil potassium, **d**) relationship between ocular dieback in the field and soil nitrogen, **e**) relationship between dieback response among inoculated plants and soil nitrogen, **f**) relationship between ocular dieback in the field and *P. cinnamomi* detection from soil baiting (+ P.C= *P. cinnamomi* detected, - P.C *P. cinnamomi* not detected), **g**) relationship between dieback response among inoculated plants and *P. cinnamomi* detection from soil baiting. Shading and error bars indicate, and solid points and lines indicate the mean and 95% confidence intervals predicted from the models, while individual points show observed dieback per plot

Following the experiment’s conclusion (187 days post-inoculation), the average dieback response among inoculated plants from different focal plant soils was marginally associated with observed dieback among plants growing in those soils in the field (*P* = 0.052) (Fig. 2a). Additionally, soil potassium and nitrogen were significant predictors of dieback among field plants and plants inoculated with field soil (Fig. 2b-e). Specifically, potassium was negatively associated with observed dieback among plants in the field (*P* = 0.032) and the dieback response among plants inoculated with field soils (*P* = 0.028). Nitrogen was positively associated with dieback in the field (*P* = 0.036) and the dieback response among plants inoculated with field soils (*P* = 0.029). Additionally, the detection of *P. cinnamomi* from soil baiting explained no significant variation in dieback among plants in the field (*P* = 0.243) or plants inoculated with field soils (*P* = 0.158) (Fig. 2f-g).

#### Influence of soil microbial community on dieback among inoculated plants

Variation partitioning revealed that dieback significantly predicted the fungal but not the bacterial community composition (Fig. S6). Specifically, the binary dieback response among inoculated plants (*P* = 0.024) and spatial (*P* = 0.001) parameters explained significant variation in the fungal community composition, while edaphic (*P* = 0.533) variables were poor predictors of fungal community composition (Fig. S6a). Contrarily, the bacterial community composition was significantly predicted by edaphic (*P* = 0.009), and spatial predictors (*P* = 0.001) whereas dieback variables were not considered in the model building process prior to partitioning variation in the bacterial community composition (Fig. S6b). CAP analysis revealed that the binary dieback response among plants inoculated with field soil (*P* = 0.005) and the spatial variables PCNM1 (*P* = 0.005) and PCNM3 (*P* = 0.030) were significant predictors of the soil fungal community (Fig. S7a), while PCNM1 (*P* = 0.005) and soil electrical conductivity (*P* = 0.005) were significant predictors of the bacterial community composition (Fig. S7b).

#### Soil microbes associated with healthy and dieback-affected inoculated plants

Indicator species analysis revealed OTUs significantly associated with healthy and dieback-affected plants inoculated with soils from either or both populations (Table 2). There were eight OTUs significantly associated with healthy plants among both populations. Notably, three *Talaromyces* OTUs (*P* = 0.019, *P* = 0.017, *P* = 0.030) were identified as significant indicators of focal plant soils where plants were healthy. *Cladophialophora* OTUs were also significant indicators of healthy plants (*P* = 0.025). Nine OTUs were significantly associated with dieback-affected plants including: *Rhodosporidiobolus* species (*P* = 0.017), *Bifiguratus* species (*P* = 0.006), and *Mortierella* species (*P* = 0.006). Ten OTUs were significantly associated with healthy plants inoculated with soil from Parr. Among these taxa, *Pisolithus* (*P* = 0.019), *Exophiala* (*P* = 0.019), and *Neophaeococcomyces* (*P* = 0.019) OTUs had the highest indicator values (Table 2). Additionally, no significant indicator taxa were associated with dieback-affected plants inoculated with soils from Parr, whereas the only significant indicator taxa from Yanderra were associated with dieback-affected plants. Of the three OTUs associated with dieback at Yanderra, one (*Exophiala* sp.) is a known pathotroph (*P* = 0.030).

**Table 2:** Indicator OTUs that were significantly associated with healthy or dieback affected *Persoonia* plants in experiment 1 (bioassay) by population. Population = ‘Both’ indicates significant indicator OTUs from a multilevel pattern analysis containing data from both populations, while Yanderra and Parr results present data from separate multilevel pattern analyses for each population. A indicates the positive predictive value (the sample estimate of the probability that the soil sample belongs to assigned group (healthy, dieback) given that the particular OTU was found), while B indicates the sensitivity (conditional probability of the species as an indicator of the assigned group (healthy, dieback)

OTU	Accession	Taxa	Group	Population	A	B	*P*	Trophic mode	Notes
ITSall_OTUb_1209	UDB0754776	*Talaromyces* sp.	Healthy	Both	0.954	0.800	0.019	Saprotroph	Pathogen antagonists (Dethoup et al. 2007; Manoch and Dethoup, 2011)
ITSall_OTUa_2109	UDB0754776	*Talaromyces* sp.	Healthy	Both	0.884	0.800	0.017	Saprotroph	Pathogen antagonists (Dethoup et al. 2007; Manoch and Dethoup, 2011)
ITSall_OTUj_13639	UDB0758162	Unidentified Herpotrichiellaceae OTU	Healthy	both	0.940	0.700	0.043	Pathotroph-Saprotroph	
ITSall_OTUj_633	UDB0620723	Unidentified Eurotiales OTU	Healthy	Both	0.931	0.700	0.018	Saprotroph	Pathogen antagonists (Dethoup et al. 2007; Manoch and Dethoup, 2011)
ITSall_OTUj_28474	UDB0754776	*Talaromyces* sp.	Healthy	Both	0.914	0.700	0.030	Saprotroph	Pathogen antagonists (Dethoup et al. 2007; Manoch and Dethoup, 2011)
ITSall_OTUe_2126	DQ008141	*Cladophialophora* sp.	Healthy	Both	0.909	0.700	0.025	Saprotroph	
ITSall_OTUj_4703	AB846969	Unidentified Umbelopsidomycetes OTU	Healthy	Both	0.886	0.700	0.022		
ITSall_OTUj_11767	UDB0756294	Unidentified Eurotiales OTU	Healthy	Both	0.883	0.700	0.030		
ITSall_OTUj_17935	AB846969	Unidentified Umbelopsidomycetes OTU	Dieback	Both	1.000	0.600	0.013		
ITSall_OTUj_2440	KY776225	Unidentified Pleosporales OTU	Dieback	Both	1.000	0.600	0.018		
ITSall_OTUj_3079	UDB059035	*Rhodosporidiobolus* sp.	Dieback	Both	1.000	0.600	0.017	Pathotroph-Saprotroph	
ITSall_OTUj_48	AB846969	Unidentified Umbelopsidomycetes OTU	Dieback	Both	0.966	0.750	0.005		
ITSall_OTUa_865	HM123225	*Bifiguratus* sp.	Dieback	Both	1.000	0.625	0.006		
ITSall_OTUc_1	HQ630337	*Mortierella* sp.	Dieback	Both	0.988	0.625	0.006	Saprotroph-Symbiotroph	
ITSall_OTUj_28265	UDB061159	Unidentified Eurotiomycetes OTU	Dieback	Both	0.971	0.625	0.006		
ITSall_OTUj_217	KC222827	Unidentified Eurotiales OTU	Dieback	Both	0.956	0.625	0.017		
ITSall_OTUa_650	JN936995	*Conlarium* sp.	Dieback	Both	0.942	0.625	0.034	Saprotroph	
ITSall_OTUa_720	AF374704	*Pisolithus* sp.	Healthy	Parr	1.000	1.000	0.019	Symbiotroph	
ITSall_OTUi_3399	MG820075	*Exophiala* sp.	Healthy	Parr	1.000	1.000	0.019	Pathotroph-Saprotroph	
ITSall_OTUm_11	KY774043	Unidentified Hymenochaetales OTU	Healthy	Parr	0.967	1.000	0.019		
ITSall_OTUa_5030	KF928484	*Neophaeococcomyces* sp.	Healthy	Parr	0.961	1.000	0.019		Some species are dark septate endophytes and known pathogen antagonists (Harsonowati et al. 2020)
ITSall_OTUj_1367	UDB0152866	Unidentified Eurotiomycetes OTU	Healthy	Parr	0.949	1.000	0.029		
ITSall_OTUj_561	KF128831	Unidentified Phaeococcomycetaceae OTU	Healthy	Parr	0.948	1.000	0.029		
ITSall_OTUa_2109	UDB0754776	*Talaromyces* sp.	Healthy	Parr	0.937	1.000	0.046	Saprotroph	Pathogen antagonists (Dethoup et al. 2007; Manoch and Dethoup, 2011)
ITSall_OTUa_4465	KM199332	*Pestalotiopsis* sp.	Healthy	Parr	0.917	1.000	0.025	Pathotroph	
ITSall_OTUi_25	JX399005	*Pestalotiopsis* sp.	Healthy	Parr	0.894	1.000	0.019	Pathotroph	
ITSall_OTUd_2161	KY523479	Unidentified Ascomycete OTU	Healthy	Parr	0.883	1.000	0.033		
ITSall_OTUj_5729	KC759484	Unidentified Hymenochaetales OTU	Dieback	Yanderra	1.000	1.000	0.030		
ITSall_OTUj_23903	UDB0731716	Unidentified Ascomycete OTU	Dieback	Yanderra	1.000	1.000	0.030		
ITSall_OTUa_4463	DQ182587	*Exophiala* sp.	Dieback	Yanderra	0.9758	1	0.030	Pathotroph	

#### Isolation of root-associated fungi

Among harvested plants, no *Phytophthora* species were reisolated from plant roots plated on *Phytophthora* selective media. Instead, other fungal taxa (*Fusarium* sp. (n = 7), *Ilyonectria* sp. (n = 7), *Cylindrocladiella* sp. (n = 2), and Ceratobasidiaceae sp. (n = 2)) were found colonizing these roots. Of the roots plated on selective media for fungi, *Penicillium* (n = 3), *Aspergillus* (n = 3), and *Trichoderma* (n = 1) were isolated (Figs. S9-S15; Table S1).

#### Relationships among above and belowground dieback responses

**Fig. 3:**
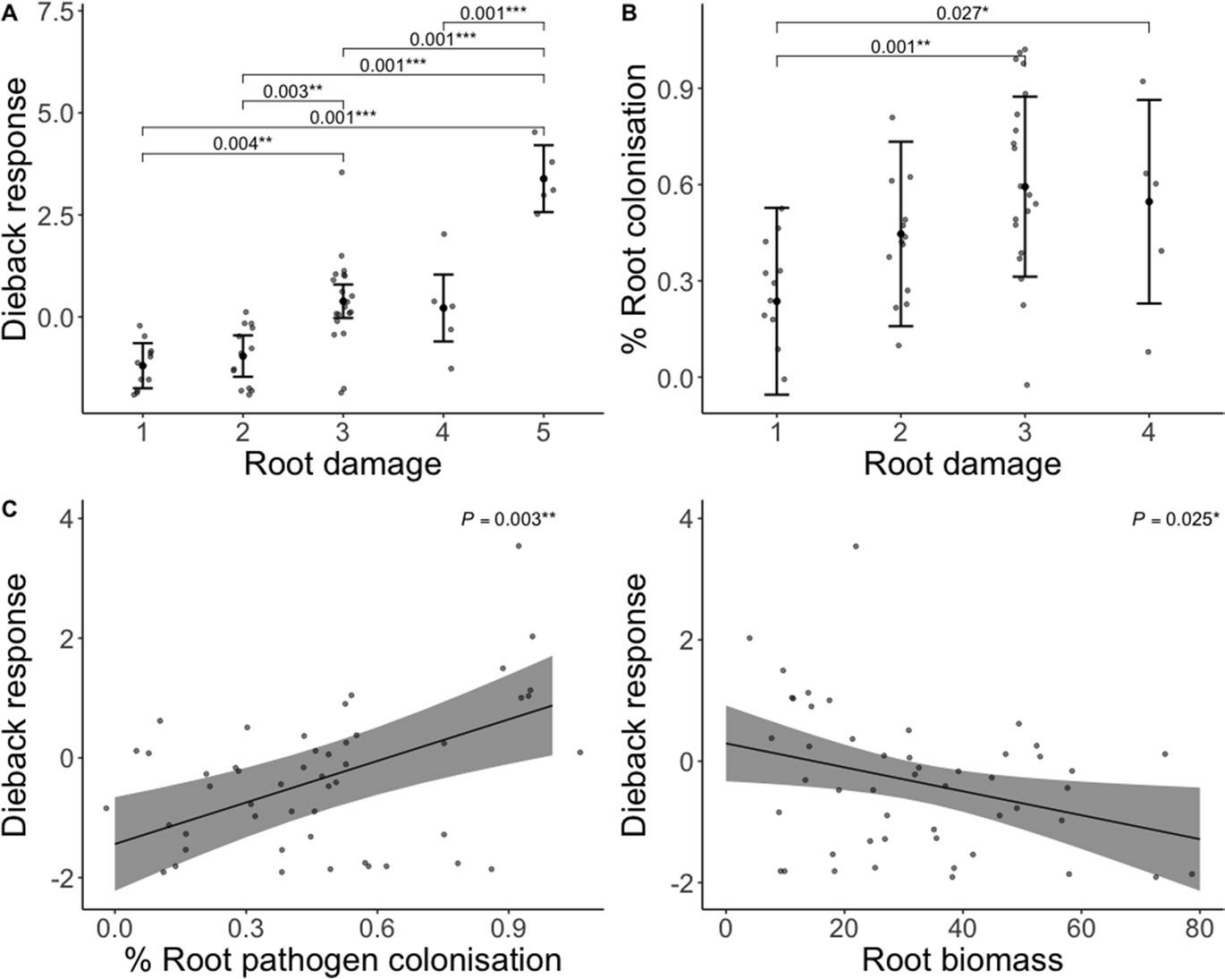
Comparisons of above and below-ground dieback among inoculated plants in experiment 1 (bioassay). **a**) Dieback response across root damage classes, **b**) proportion of roots with colonisation on *Phytophthora*-selective media across root damage classes (root damage class 5 was not included in these analyses for no living roots were recovered from plants in this damage class), **c**) Effect of proportion of roots with colonisation on *Phytophthora*-selective media on dieback response, **d**) effect of root biomass on dieback response. Brackets above groups in panels A and B indicate significant pairwise comparisons with *P* values

Dieback response significantly varied among different levels of root damage (*P* < 0.001) (Fig. 3a). Specifically, above ground dieback response was highest in the highest root damage class and generally decreased among the lower root damage classes (Fig. 3a). The proportion of roots plated on *Phytophthora* selective media with fungal colonization also varied among different levels of root damage (*P* = 0.002), with the highest percent fungal colonization observed among root classes 3 and 4 (root damage class 5 was not included in these analyses since no living roots were recovered from plants in this damage class) (Fig. 3b). Additionally, dieback response was positively associated with root pathogen colonization (*P* = 0.003) (Fig. 3c) and negatively associated with root biomass (*P* = 0.025) (Fig. 3d). Finally, dieback response showed no apparent relationship with above ground biomass (*P* = 0.575).

#### Post-harvest culture identification and in vitro dual culture antagonism assay

**Fig. 4:**
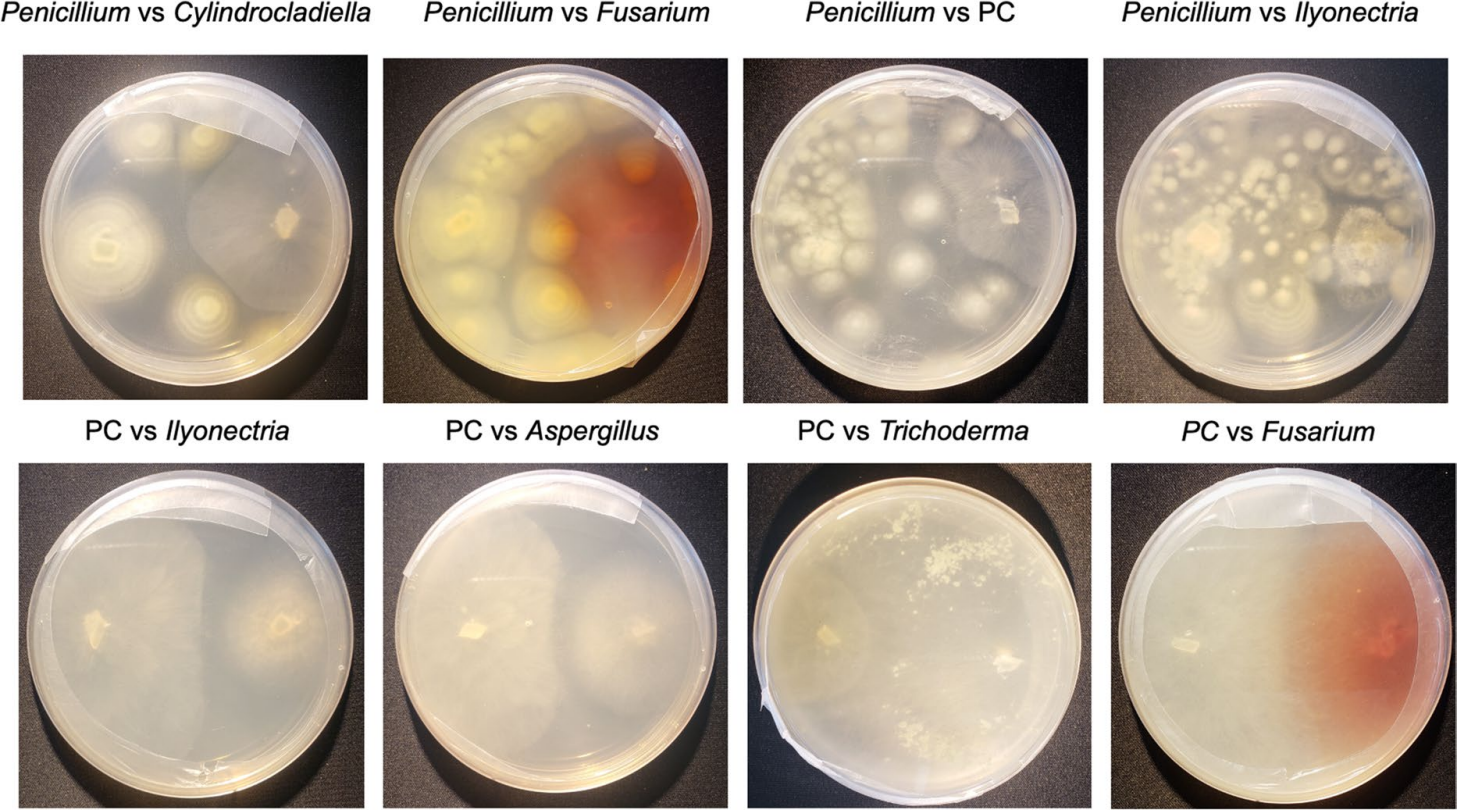
Photographs of isolates from in vitro dual culture assay, after 9 days of growth of malt extract agar, where native fungal isolates from *P. hirsuta* roots and *Phytophthora cinnamomi* (PC) baited from soil were placed in competition. Labels indicate position of cultures in the below photos (left vs right)

The in vitro dual culture competition assay revealed several antagonistic interactions among fungi isolated from *P. hirsuta* roots and *P. cinnamomi* isolated from *P. hirsuta* rhizosphere soil (Fig. 4). The *Trichoderma* sp. (*P* = 0.016), *Aspergillus* sp. (*P* = 0.021), *Penicillium* sp. (*P* = 0.023), and *Ilyonectria* sp. (*P* = 0.042) cultures exhibited significant quantitative antagonism against *P. cinnamomi* (Fig. 4). Among these cultures, qualitative antagonism against *P. cinnamomi* was also observed in the form of inhibition or overgrowth. Additionally, *Fusarium* sp. exhibited marginal quantitative antagonism (*P* = 0.059) and qualitative overgrowth on *P. cinnamomi* when placed in dual culture. In addition to showing antagonism against *P. cinnamomi, Trichoderma* sp. showed quantitative antagonism against *Fusarium* sp. (*P* = 0.036) and *Cylindrocladiella* sp. (*P* = 0.034). *Ilyonectria* sp. growth was strongly quantitatively and qualitatively inhibited when grown in dual culture alongside *Penicillium* sp. (*P* = 0.024) and *Cylindrocladiella* sp. growth was strongly qualitatively inhibited when grown in dual culture alongside *Aspergillus* sp.

### Experiment 2 (Nitrogen addition)

#### Effect of nitrogen addition on observed measures of dieback and plant growth

**Fig. 5:**
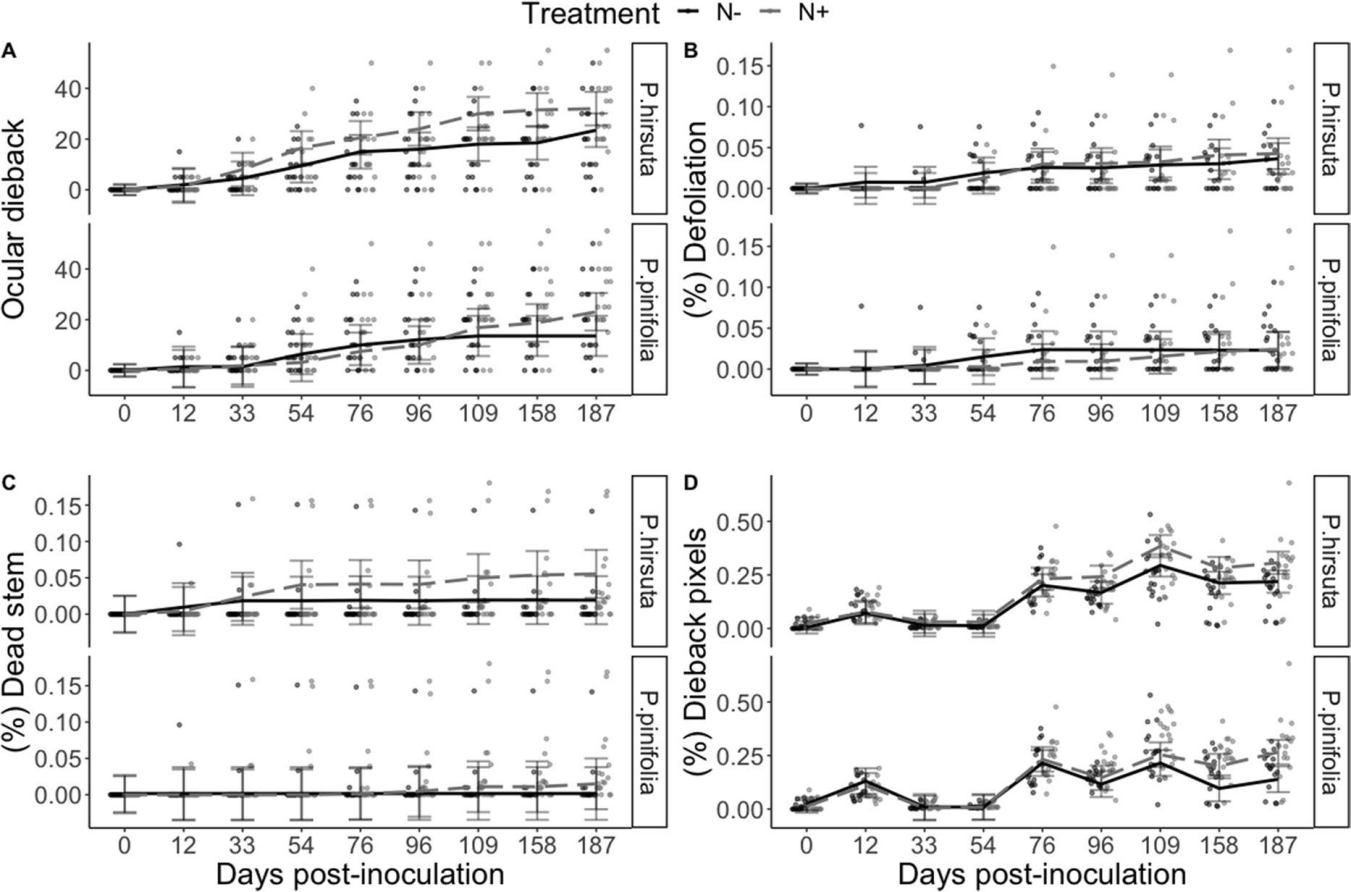
Dieback response over time by species and ambient (N-) or elevated (N+) nitrogen addition. **a**) Ocular estimated dieback response, **b**) proportion of stem death relative to total plant height, **c**) proportion of dieback pixels relative to healthy pixels, **d**) relative growth rate. Error bars indicate the mean and 95% confidence intervals predicted from the mixed model, while individual points show observed data

Ocular dieback significantly varied over time since inoculation (*P* < 0.001), and by treatment (*P* < 0.001), which was further modified by species (*P*_N:Species_ = 0.024) (Fig. 5a). On average, plants treated with elevated N had 3.44% more dieback than plants under ambient N (*P* = 0.002). Specifically, ocular dieback was 6.33% higher for *P. hirsuta* plants under elevated N compared to ambient N (*P* < 0.001), whereas there was no significant effect of nitrogen addition on ocular dieback for *P. pinifolia* plants (*P* > 0.05). Irrespective of treatment, ocular dieback was 6.51% higher for *P. hirsuta* plants compared to *P. pinifolia* plants (*P* < 0.001). The proportion of defoliation relative to total plant height significantly varied over time (*P* = 0.001), and species (*P* = 0.012) with defoliation relative to total plant height 0.8% higher for *P. hirsuta* plants compared to *P. pinifolia* plants (*P* = 0.014) (Fig. 5b). However, defoliation was not observed to vary between N treatments (*P* > 0.05). The proportion of stem death relative to total plant height significantly varied over time (*P* = 0.031), and also by treatment (*P* = 0.002), which was marginally modified by species (*P*_N:Species_ = 0.049) (Fig. 5c). On average, *P. hirsuta* plants with elevated N had 1.9% more stem death than plants with ambient N (*P* < 0.001), while there was no significant effect of nitrogen addition on stem death for *P. pinifolia* plants (*P* > 0.05). Additionally, *P. hirsuta* plants with elevated N had 2.96% more stem death than *P. pinifolia* plants with elevated N (*P* < 0.001), and 1.40% higher stem death under ambient N (*P* = 0.014). The proportion of dieback pixels relative to healthy pixels significantly varied over time since inoculation (*P* < 0.001), which was further modified by treatment (*P*_N:Time = 0.006) and species (*P*_N:Species_ < 0.001) (Fig. 5d). On average, the proportion of dieback pixels relative to healthy pixels was 3.83% higher for plants with elevated N compared to ambient N (*P* < 0.001); however, this effect was only observed from 96 days post-inoculation until the final monitoring event. Additionally, the proportion of dieback pixels relative to healthy pixels was 3.42% higher for *P. hirsuta* compared to *P. pinifolia* (*P* < 0.001), which was also observed from 96 days post-inoculation onward (Fig. 5d).

#### Experimental harvest

**Fig. 6:**
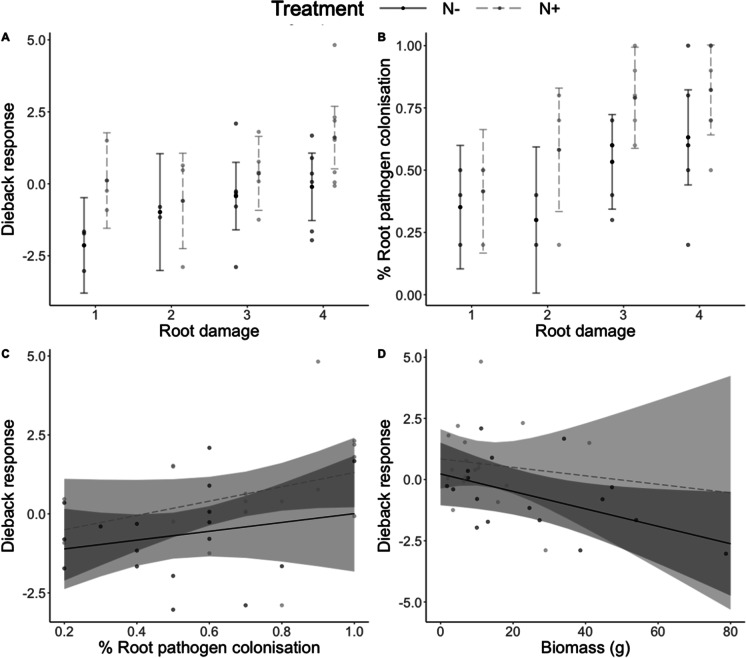
Comparisons of above and below-ground dieback among inoculated plants in experiment 2 (nitrogen addition) by treatment. **a**) Dieback response across root damage classes and N treatment, **b**) proportion of plated roots with colonisation on *Phytophthora*-selective media across root damage classes and N treatment, **c**) effect of root pathogen colonisation and N treatment on dieback response, **d**) effect of root biomass and N treatment on dieback response

Nitrogen treatment (*P* = 0.013) significantly predicted dieback response (Fig. 6a). Specifically, dieback response was 1.28 times higher among plants with elevated N compared to ambient (*P* = 0.025) (Fig. 6a). Additionally, root damage (*P* = 0.063) marginally predicted dieback response and increased with increasing dieback. However, the effect of nitrogen addition on dieback response was not further modified by observed root damage (*P* = 0.642). Root damage (*P* = 0005) and nitrogen treatment (*P* = 0.006) also significantly predicted the proportion of roots plated on *Phytophthora* selective media with fungal colonization by probable pathogens (Fig. 6b). On average, elevated N increased probable pathogen colonization by 19.8% (*P* = 0.012) (Fig. 6b). Additionally, the proportion of root colonization increased over higher levels of root damage, with 34.3% higher colonization observed in root damage class 4 compared to 1 (*P* = 0.008), and 27.8% higher colonization observed in root damage class 3 compared to 1 (*P* = 0.051) (Fig. 6b). However, the effect of nitrogen treatment on root colonization was not further modified by root damage (*P* = 0.768). Dieback response showed no apparent relationship with root pathogen colonization (*P* = 0.130) or treatment (*P* = 0.115) among plants in the N experiment (Fig. 6c). Additionally, dieback response was negatively associated with root biomass, but the effect was only marginal (*P* = 0.076) and showed no relationship with nitrogen treatment (*P* = 0.123) (Fig. 6d).

## Discussion

The results from these experiments suggest soil pathogens may be involved in driving dieback in *Persoonia,* but the effect is contextual in that it may be further modified by other abiotic and biotic characteristics of the soil environment. For example, we identified soil fungi which were significant indicators of healthy inoculated plants and root-associated fungi that possess antagonistic qualities to known pathogens. Additionally, we found that variation in abiotic properties of the soil environment such as nitrogen and potassium significantly influenced dieback in the field and in these experiments.

Many pathogen-associated plant dieback etiologies involve interactions among multiple pathogens that impact host fitness and complicate the appropriate diagnosis of underlying dieback drivers (Abdullah et al. 2017; Lamichhane and Venturi 2015). Although *P. cinnamomi* was frequently isolated from field soils at both populations, we observed other known pathogens (*Fusarium*, *Ilyonectria* and *Cylindrocladiella*) in the roots of plants inoculated with field soils. In other systems, these pathogens have been identified as secondary pathogens that impact roots already damaged by *Phytophthora* (Aghighi et al. 2014) and increase in abundance in the soil when *Phytophthora* is present (Solís-García et al. 2021) which could explain why these taxa were frequently isolated from roots on the pathogen-specific media following harvest.

Additionally, *Fusarium* and *Ilyonectria* exhibited some antagonistic qualities against *P. cinnamomi* when placed in dual culture which could explain why these pathogens were more frequently isolated from plant roots. Although this study lacks strong evidence for *P. cinnamomi* as a dieback driver, further work is required before the possibility is ruled out. Since the experiment was conducted as a pot study with field soils, it is possible that additional factors associated with this experiment such as reduced watering frequency, or the absence of environmental stressors present in the field might have prevented *P. cinnamomi* colonization and infection. Thus, we conclude that both *P. cinnamomi* and the other pathogens detected in this study warrant further investigation. Subsequent studies could aim to characterize symptoms of disease and susceptibility to dieback in *P. hirsuta* by inoculating plants with combinations of these candidate dieback-driving pathogens to disentangle the possible role of a co-infection network (Belisario et al. 2004; Dung et al. 2014; Sagar and Sugha 1997).

Many fungi can suppress the growth of plant pathogens and onset of disease, illustrating their utility in managing dieback in natural and agricultural ecosystems (Chet and Inbar 1994; Thambugala et al. 2020). This study identified several soil-associated fungi that were indicators of healthy plants and root-associated fungi that possess pathogen antagonistic capabilities. For example, *Talaromyces* species are known microbial antagonists to phytopathogenic fungi and were identified as significant indicators in the soil where plants were not exhibiting dieback symptoms (Dethoup et al. 2007; Manoch and Dethoup 2011). Additionally, *Trichoderma*, *Aspergillus* and *Penicillium* species were frequently isolated from plant roots and exhibited antagonistic capabilities against *P. cinnamomi*, which has also been observed in past studies (Chambers and Scott 1995; Downer et al. 2001; Malajczuk et al. 1977). *Penicillium* species also exhibited strong antagonism to the other three pathogenic fungi isolated from *Persoonia* roots (*Fusarium*, and *Ilyonectria* and *Cylindrocladiella*). Consistent with these results, past studies have found a competitive advantage of *Penicillium* species against *Fusarium* species in dual culture (Win et al. 2021) and have used *Penicillium* isolates in the field to control disease (De Cal et al. 2009; Larena et al. 2003; Li et al. 2019). However, it should also be mentioned that many of the fungal isolates which displayed antagonistic qualities to known pathogen taxa have also been attributed to plant disease in other pathosystems (Louw & Korsten 2014; Pawar et al. 2008). In future, more effort should be taken to identify these isolates to species (Figs. S8-S15) and screen for their attribution to disease and utility against pathogen drivers of dieback (Mejía et al. 2008; Raymaekers et al. 2020).

Soil physiochemical characteristics are important drivers of many regulatory processes for both plants and microbes and significantly influence plant defense and disease susceptibility (Anothai and Chairin 2020; Orr and Nelson 2018). In the first experiment, total soil nitrogen was positively associated with field and inoculated plant dieback, and plants in the second experiment exhibited higher dieback and higher root colonization by probable pathogens when treated with ammonium nitrate. Nitrogen nutrition is important in many plant and pathogen biological processes and has long been hypothesized as a driver of dieback in forest ecosystems by impacting plant physiological mechanisms and encouraging infection by pathogenic fungi (Jurskis and Turner 2002; Nihlgård 1985; Scarlett et al. 2013). However, the role of nitrogen nutrition in plant defense and pathogen virulence is still poorly understood for many pathosystems (Sun et al. 2020). For example, significantly greater dieback in experiment two was observed among *P. hirsuta* individuals treated with ammonium nitrate compared to *P. pinifolia* individuals of the same treatment. Consistent with these results, Scarlett et al. (2013) also observed species-specific variation in susceptibility to soil pathogens under the same nitrogen treatment. If *P. pinifolia* is less susceptible than *P. hirsuta* to pathogenic fungi under elevated ammonium nitrate this might help explain why *P. pinifolia* is more widespread and abundant in the landscape, yet this still does not explain why greater dieback was observed among *P. pinifolia* plants with higher total soil nitrogen in experiment 1. Additionally, nitrogen addition in experiment 2 resulted in higher root colonization on pathogen selective media; however, these results suggest that nitrogen addition may accelerate plant dieback response irrespective of potential pathogen loads (Fig. 6c). If this is the case, managing soil nitrogen levels in the soil may be a useful tool to manage dieback in the landscape. Furthermore, the small effect of nitrogen addition on observed dieback responses in experiment 2 suggests that further work is required to explore this relationship as plant responses may differ under varying conditions in a field setting (Limpens et al. 2012; Poorter et al. 2016). While both studies have found an important relationship among soil nitrogen and dieback, further work is needed to explore how nitrogen nutrition impacts dieback in *Persoonia* both in the presence and absence of pathogens, to understand the role of nitrogen nutrition and pathogen susceptibility as contributory or causal agents of dieback. In the first experiment, a negative relationship was also observed among field and inoculated plant dieback under increasing soil potassium. In a review of published studies on potassium nutrition and plant disease, Perrenoud (1990) found that potassium fertilization reduced the incidence of diseases caused by fungal pathogens by 70%. Potassium fertilization is also known to assist plants in the synthesis of defensive compounds, induce the thickening of cells walls, and repair of damaged tissue (Mengel and Kirkby 2001; Sarwar, 2012; Wang et al. 2013). It is possible that potassium fertilization could be applied to reduce dieback symptoms in the field (Grewal and Williams 2002; Rodrigues et al. 2009). However, further study into the role of potassium nutrition in alleviating plant stress and symptoms of dieback in *Persoonia* is warranted to determine its efficacy as a management action.

Investigating the role of soil variation on plant dieback with relevance to the field is a critical step to uncover edaphic mechanisms driving dieback in native plants and designing management plans to mitigate disease. The results from these experiments suggest that soil abiotic variation and the presence of soil pathogens may contribute to dieback in *Persoonia*. The results from this study have also identified native root-associated fungi with pathogen inhibiting properties and dieback inhibition under elevated soil potassium which could be implemented into research and management efforts to reduce dieback in the field. These findings have important implications for the ongoing conservation and management of this Endangered shrub and serves as a model case study for future research addressing edaphic drivers of dieback in native landscapes.

## Supplementary Information

ESM 1 (DOCX 43553 kb)

## Data Availability

The datasets generated from these experiments and scripts used for analyses are available from the corresponding author at the following link https://osf.io/smukz/. Raw DNA sequencing (Illumina) data are available under NCBI BioProject IDs PRJNA861111 and PRJNA861120. Sequences obtained from cultured isolates from this study are available under the NCBI GenBank submission ID SUB11835494.
