# Supplementary material for: Soil chemistry and fungal communities are associated with dieback in an Endangered Australian shrub
Source: Plant Soil. 2022 Oct 1;483(1-2):47–70. doi: 10.1007/s11104-022-05724-7 (PMC9525234; doi:10.1007/s11104-022-05724-7)
Supplement: Supplementary file 1 — (DOCX 43553 kb) [file 11104_2022_5724_MOESM1_ESM.docx]

**Supplementary information**

**Table S1** List of isolates collected from focal plant soil baiting and post-harvest root culturing. Identification of isolates was based on closest match (query cover and % identity) to isolates from the NCBI database. Phylogenetic tree ID corresponds to the labels used to differentiate isolates from this study in the phylogenetic trees (Figures S8-S15). The sequenced isolates from this study can be found via the accession numbers in this table at the NBCI GenBank (<http://blast.ncbi.nlm.nih.gov>).

| **Taxon** | **Soil code** | **Phylogenetic tree ID** | **Soil baiting** | **Post-harvest root culturing** | **Antagonism assay** | **GenBank accession** |
| --- | --- | --- | --- | --- | --- | --- |
| *Aspergillus* sp. | Parr 3 | Aspergillius_isolate_1 |  | 1 |  | OP035330 |
| *Aspergillus* sp. | Parr 3 | Aspergillius_isolate_2 |  | 1 |  | OP035331 |
| *Aspergillus* sp. | Parr 3 | Aspergillius_isolate_3 |  | 1 | 1 | OP035332 |
| Ceratobasidiaceae | Yan 9 | Ceratobasidiaceae_isolate_1 |  | 1 |  | OP035333 |
| *Ceratobasidium* sp. | Yan 2 | Ceratobasidium_isolate_1 |  | 1 |  | OP035334 |
| *Cylindrocladiella* sp. | Yan 2 | Cylindrocladiella_isolate_1 |  | 1 |  | OP035335 |
| *Cylindrocladiella* sp. | Yan 2 | Cylindrocladiella_isolate_2 |  | 1 | 1 | OP035336 |
| *Fusarium* sp. | Yan 9 | Fusarium_isolate_1 |  | 1 |  | OP035337 |
| *Fusarium* sp. | Yan 8 | Fusarium_isolate_2 |  | 1 | 1 | OP035338 |
| *Fusarium* sp. | Yan 1 | Fusarium_isolate_3 |  | 1 |  | OP035339 |
| *Fusarium* sp. | Parr 5 | Fusarium_isolate_4 |  | 1 |  | OP035340 |
| *Fusarium* sp. | Parr 5 | Fusarium_isolate_5 |  | 1 |  | OP035341 |
| *Fusarium* sp. | Parr 5 | Fusarium_isolate_6 |  | 1 |  | OP035342 |
| *Fusarium* sp. | Parr 5 | Fusarium_isolate_7 |  | 1 |  | OP035343 |
| *Ilyonectria* sp. | Parr 2 | Ilyonectria_isolate_1 |  | 1 |  | OP035344 |
| *Ilyonectria* sp. | Parr 5 | Ilyonectria_isolate_2 |  | 1 | 1 | OP035345 |
| *Ilyonectria* sp. | Yan 1 | Ilyonectria_isolate_3 |  | 1 |  | OP035346 |
| *Ilyonectria* sp. | Yan 2 | Ilyonectria_isolate_4 |  | 1 |  | OP035347 |
| *Ilyonectria* sp. | Yan 1 | Ilyonectria_isolate_5 |  | 1 |  | OP035348 |
| *Ilyonectria* sp. | Yan 2 | Ilyonectria_isolate_6 |  | 1 |  | OP035349 |
| *Ilyonectria* sp. | Parr 8 | Ilyonectria_isolate_7 |  | 1 |  | OP035350 |
| *Penicillium* sp. | Parr 2 | Penicillium_isolate_1 |  | 1 | 1 | OP035352 |
| *Penicillium* sp. | Parr 3 | Penicillium_isolate_2 |  | 1 |  | OP035351 |
| *Penicillium* sp. | Parr 3 | Penicillium_isolate_3 |  | 1 |  | OP035353 |
| *Phytophthora cinnamomi* | Parr 8 | Phytophthora_isolate_1 | 1 |  |  | OP035360 |
| *Phytophthora cinnamomi* | Parr 4 | Phytophthora_isolate_2 | 1 |  |  | OP035354 |
| *Phytophthora* sp. | Parr 2 | Phytophthora_isolate_3 | 1 |  |  | OP035355 |
| *Phytophthora cinnamomi* | Parr 5 | Phytophthora_isolate_4 | 1 |  | 1 | OP035356 |
| *Phytophthora cinnamomi* | Yan 3 | Phytophthora_isolate_5 | 1 |  |  | OP035357 |
| *Phytophthora cinnamomi* | Parr6 | Phytophthora_isolate_6 | 1 |  |  | OP035362 |
| *Phytophthora* sp. | Parr 1 | Phytophthora_isolate_7 | 1 |  |  | OP035358 |
| *Phytophthora cinnamomi* | Yan 7 | Phytophthora_isolate_8 | 1 |  |  | OP035359 |
| *Phytophthora cinnamomi* | Yan 6 | Phytophthora_isolate_9 | 1 |  |  | OP035361 |
| *Trichoderma* sp. | Yan 9 | Trichoderma_isolate_1 |  | 1 | 1 | OP035363 |

**Table S2** Mean and standard deviation (SD) of dieback observed, and the soil parameters measured across the two sites (Parr, Yanderra).

| Population | Dieback | Phosphorus | Calcium | Magnesium | Total Carbon | Total Nitrogen | pH | Available water capacity |
| --- | --- | --- | --- | --- | --- | --- | --- | --- |
| Unit | (%) | (mg/kg) | (%) | (%) | (%) | (%) |  | (%) |
| Parr | 21.111 | 0.12 | 35.067 | 27.334 | 1.102 | 0.044 | 4.356 | 0.264 |
| SD | 14.53 | 0.92 | 13.152 | 8.118 | 0.587 | 0.018 | 0.175 | 0.035 |
| Yanderra | 21.667 | 0.17 | 17.812 | 17.882 | 3.368 | 0.09 | 4.539 | 0.305 |
| SD | 11.726 | 2.225 | 6.995 | 5.558 | 1.733 | 0.045 | 0.161 | 0.1 |

**
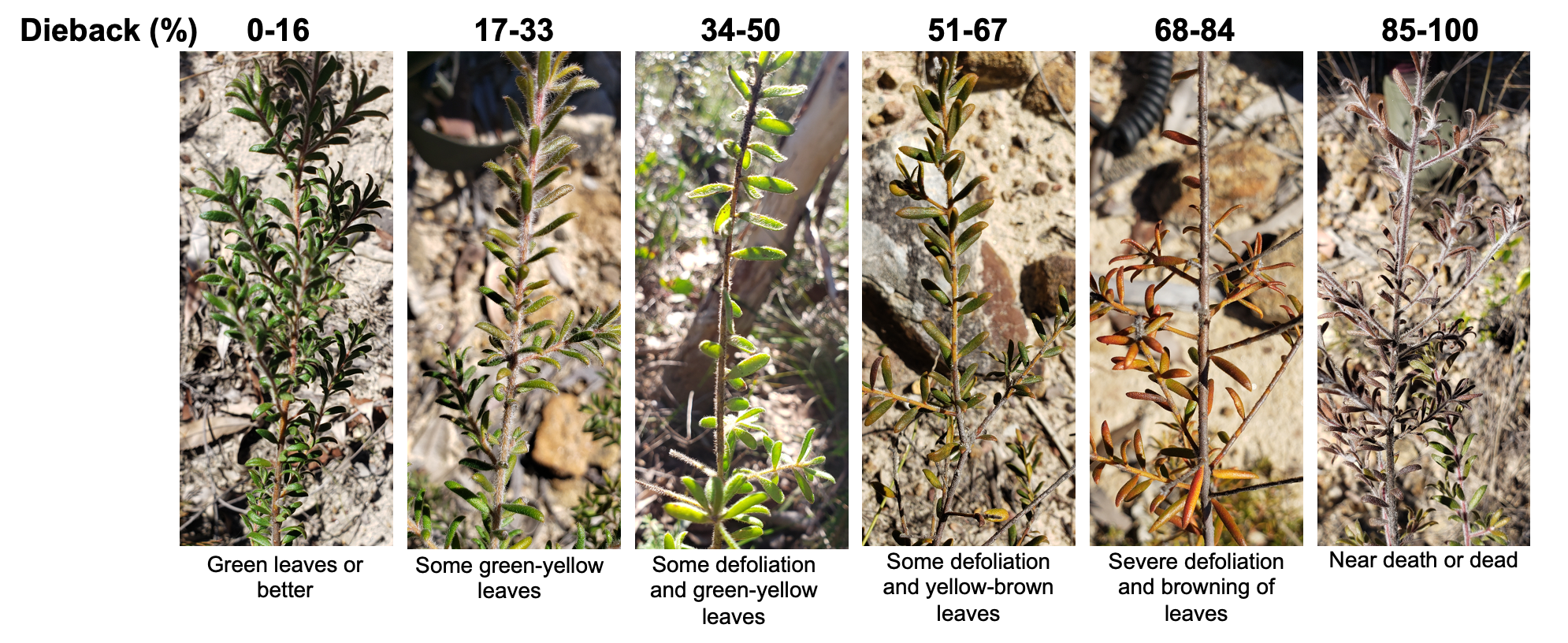
**

**Figure S1** Description of metrics used to determine dieback among *Persoonia hirsuta* seedlings in the field. Seedling dieback was scored subjectively by signs of defoliation, leaf discoloration, and stem death and the proportion of the plant exhibiting these symptoms. Images show examples of *P. hirsuta* individuals that would fall into different dieback groupings. Adapted from: Andres S. E., Powell J. R., Gregory D., Offord C. A. & Emery N. J. Assessing translocation management techniques through experimental trials: a case study of the endangered shrub *Persoonia hirsuta*. (2021). *Restoration Ecology* doi: <https://doi.org/10.1111/rec.13603>. e13603. Health classes have been removed from the figure in this adaptation


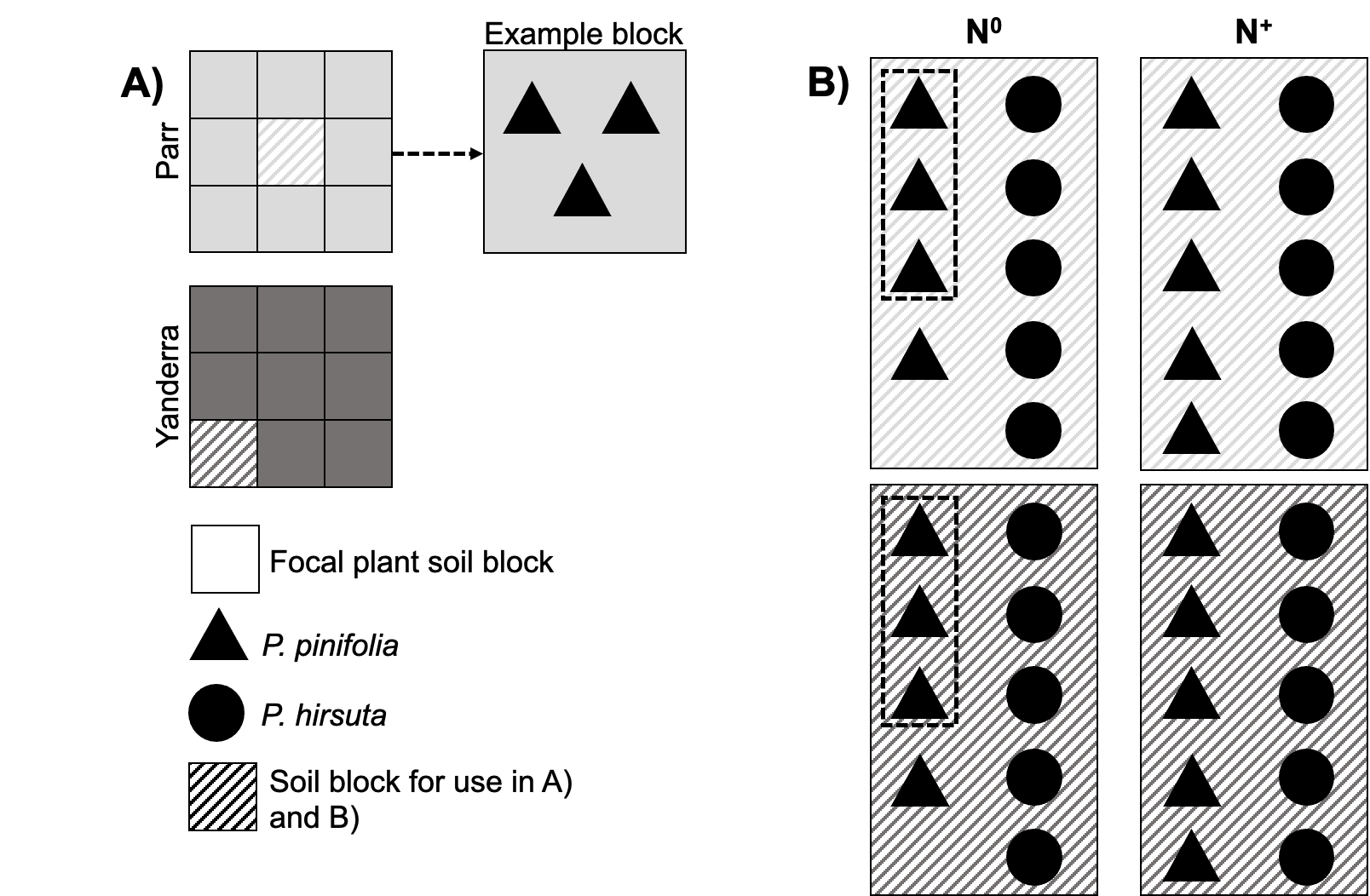


**Figure S2** a) Bioassay experimental study design. 18 plots with soils from 9 focal plants at 2 populations (Yanderra and Parr). Each plot consists of 3 replicate *P. pinifolia* plants. Diagonal lines indicate the 2 focal plant soils (1 from each population) to be included in the nitrogen addition experiment. b) Nitrogen addition experimental design (soils from 2 focal plants in A)). N^0^ 4 *P. pinifolia* plants and 5 *P. hirsuta* plants grown in soils from each focal plant at Yanderra and Parr under ambient soil nitrogen. Dashed line around 3 *P. pinifolia* plants in N^0^ indicate 3 plants from a) that will be included in this monitoring dataset (the additional *P. pinifolia* plant in N^0^ is for additional experimental replication only to be included in study b)). N^+^ 5 *P. pinifolia* and 5 *P. hirsuta* plants grown in the same focal plant soil at N^0^ with a mixture 100 mL of a 0 mg L^-1^ or 100 mg L^-1^ solution of ammonium nitrate added.

**Figure S3** a-b) Experimental setup. c) Repeated photography monitoring setup. d) Example photo of how repeated photos were taken.


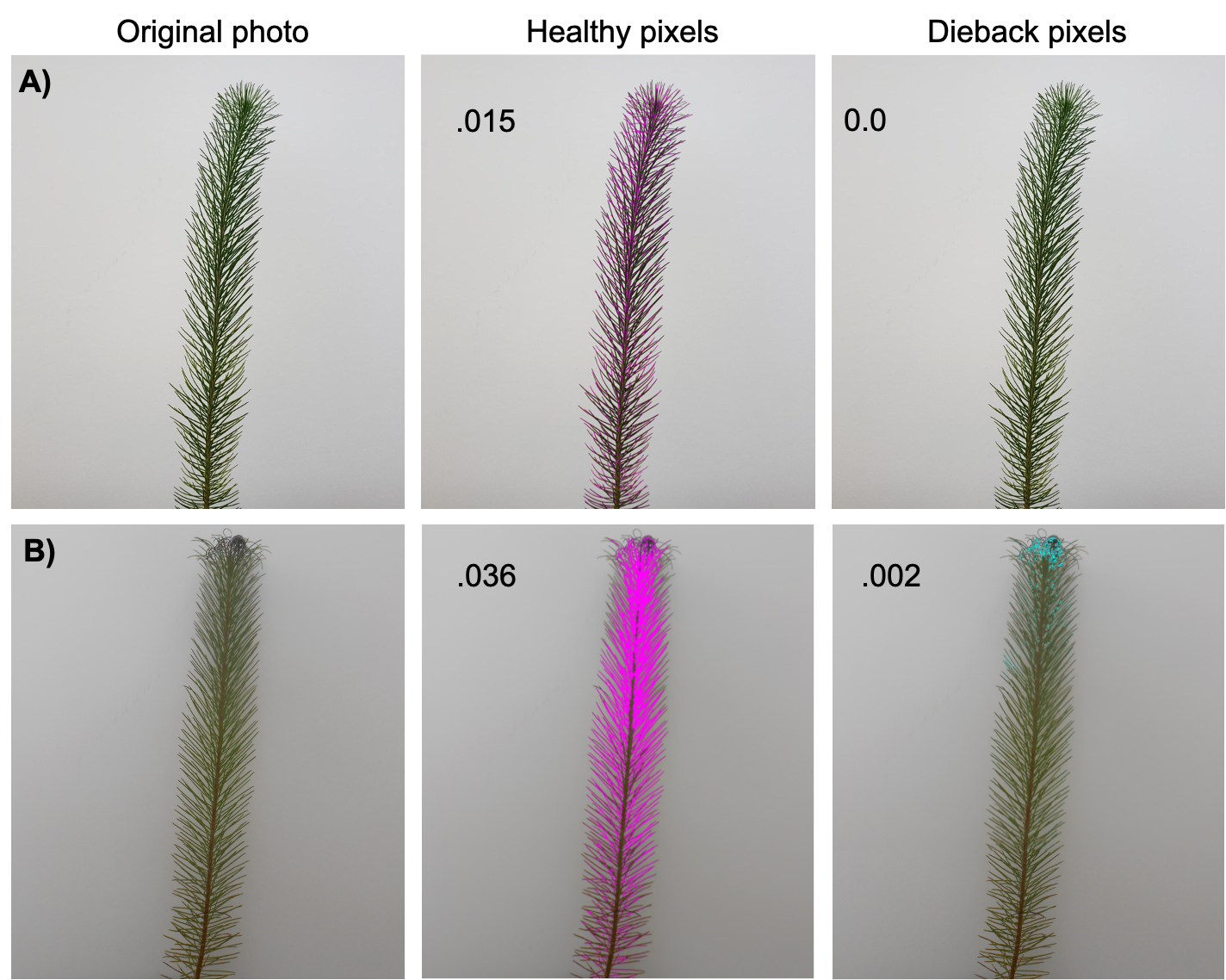


**Figure S4** Examples of repeat photos for two plants a) and b) with overlaid color margins for healthy (pink) and dieback (blue) pixels, numbers correspond to the extracted values associated with the image analysis output (a=0% dieback pixels, b=0.056% dieback pixels). Healthy pixels were centered using a RGB triplet of 0.25, 0.45, 0.25 and a radius of 0.2. Dieback pixels were centered using RGB triplets for dead (black) plant material (0.2, 0.2, 0.1), and discoloration (yellow) plant material (0.23, 0.25, 0) with a radius of 0.02 and 0.03 respectively.


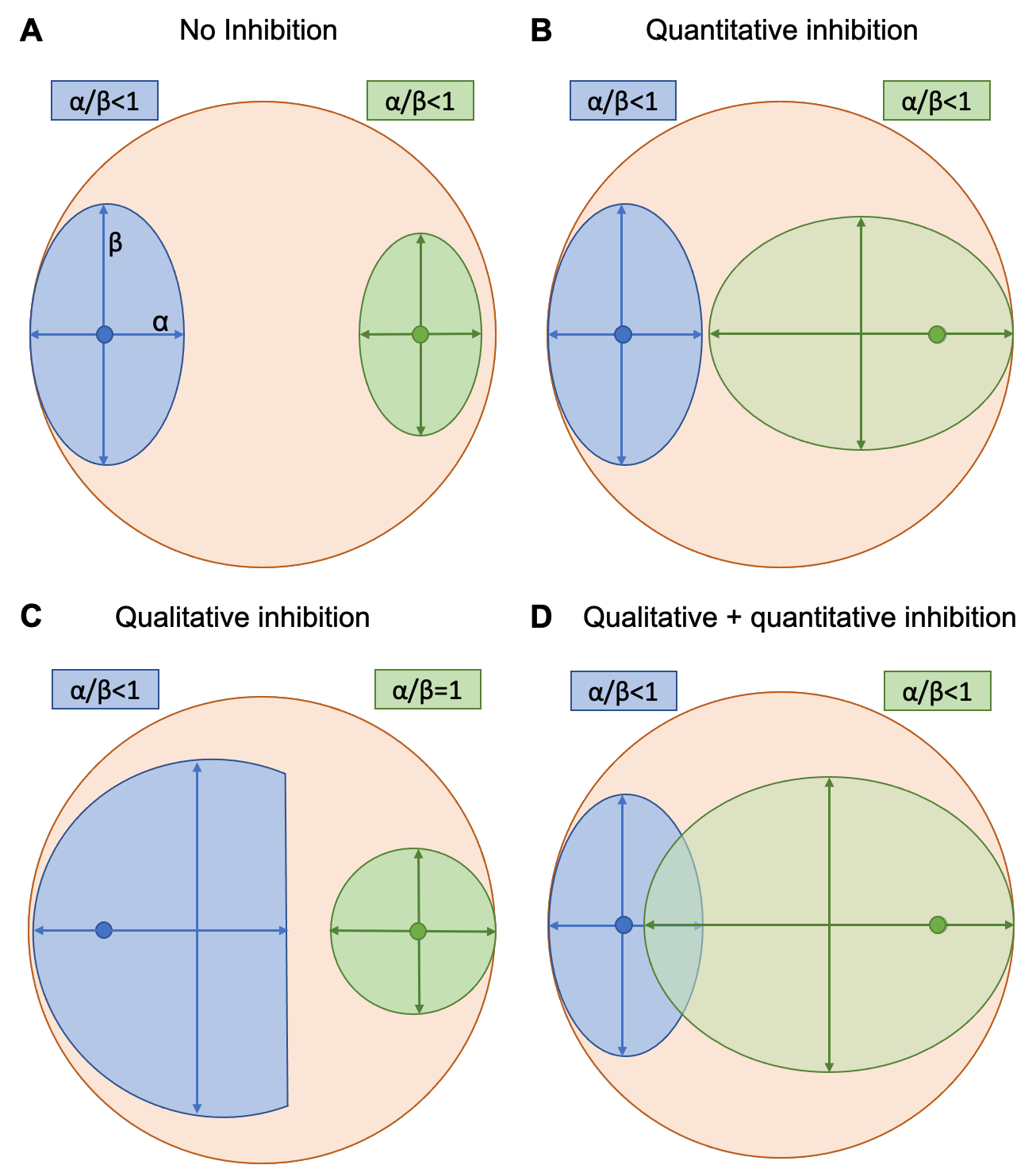


**Figure S5** Schematic representation of example dual culture antagonism plates among two cultures (blue and green) with the major (α) and minor (β) axes of growth labelled; a) plate with neither quantitative nor qualitative inhibition among the two isolates, b) plate representing quantitative inhibition observed for the blue isolate, c) a plate representing qualitative inhibition for the blue isolate (inhibition zone), d) a plate representing qualitative (overgrowth) and quantitative inhibition for the blue isolate.


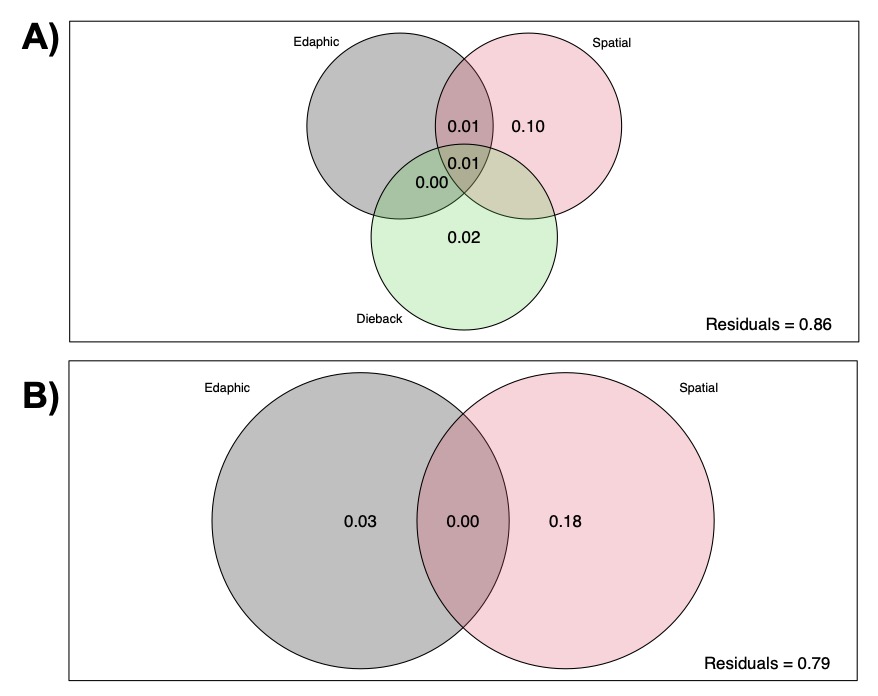


**Figure S6** Variation partitioning was performed to investigate drivers of a) fungal, and b) bacterial structure in the soil based on dieback, edaphic, and spatial factors.


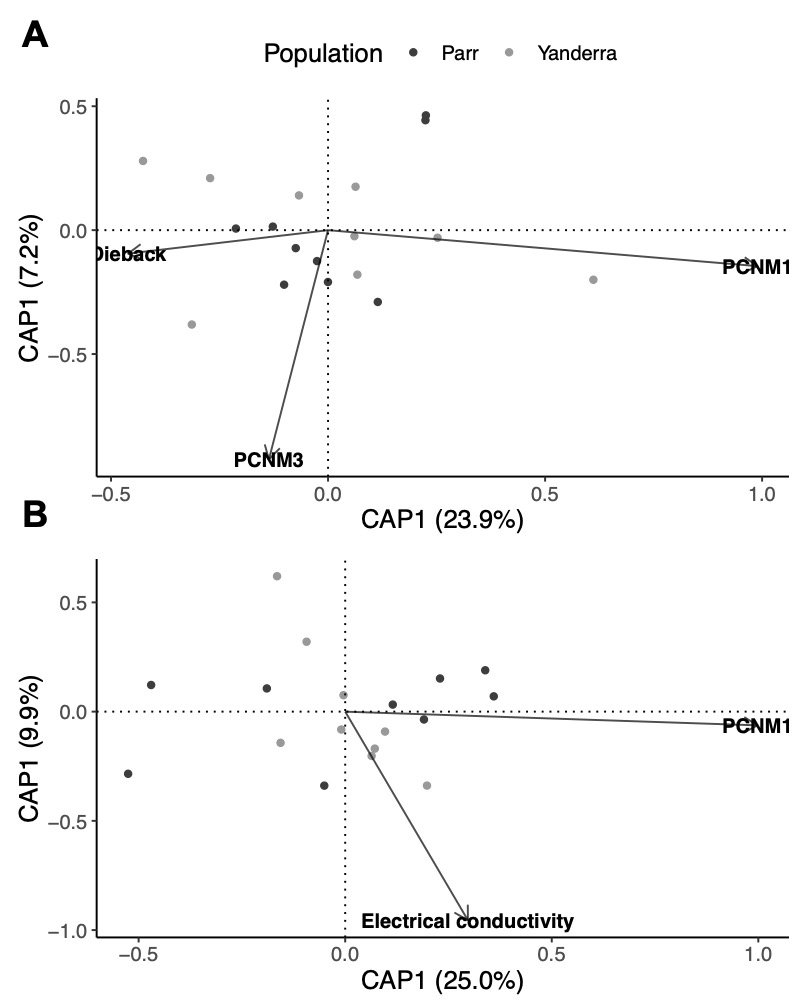


**Figure S7** Soil microbial communities from Yanderra and Parr. Constrained Analysis of Principal Coordinates (CAP) ordination of (a) fungal and (b) bacterial communities. Plots were constrained by significant edaphic, dieback, and spatial variables obtained from variation partitioning. Only significant variables that explained total variation were plotted. PCNM1 and PCNM3 relate to the first and third axis extracted from Principal Coordinates of Neighbour Matrices extracted from the spatial location of plots where soils were sampled in the field.


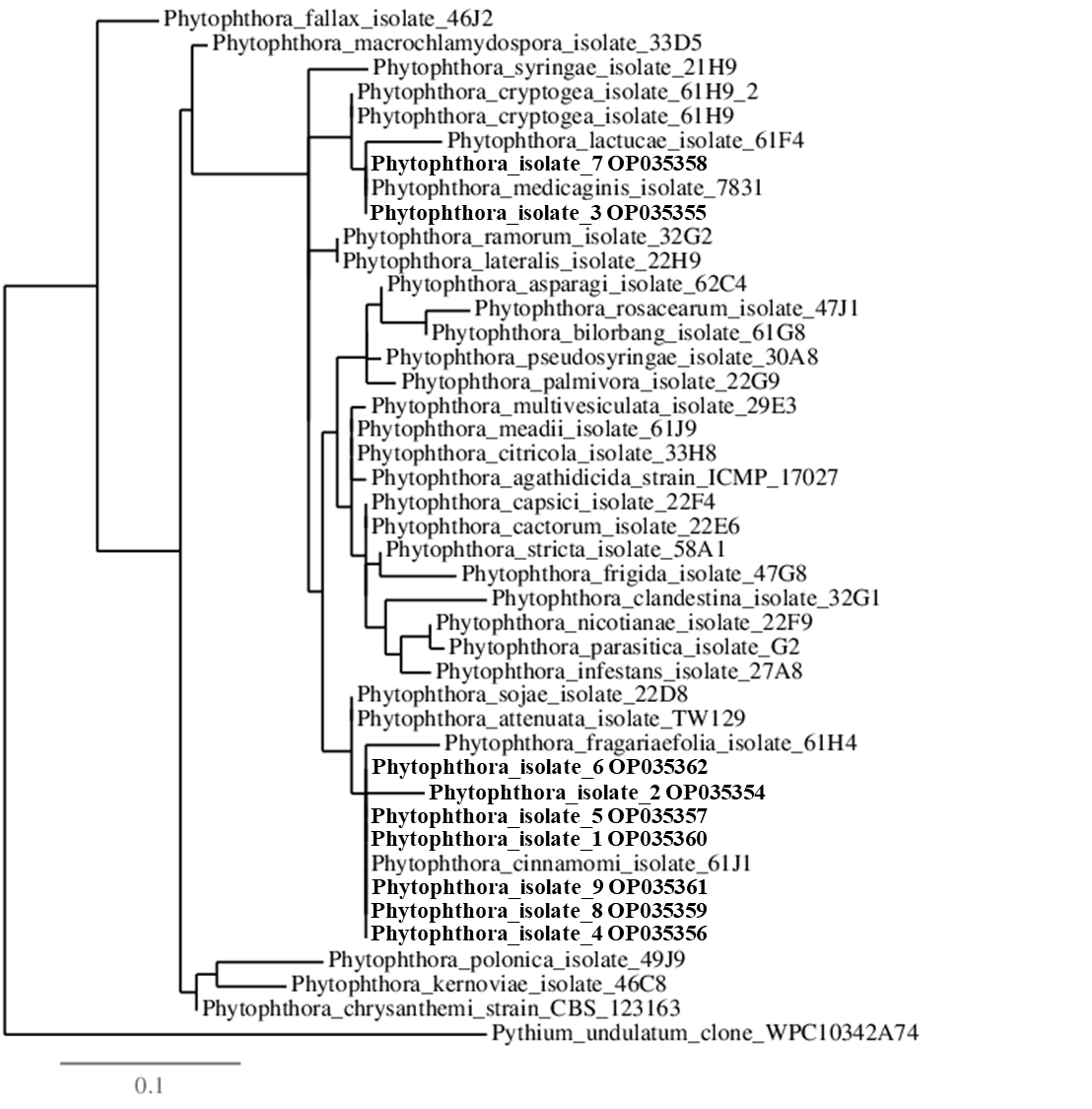


**Figure S8** Tree based on ITS sequences showing the phylogenetic position of isolates obtained in this study in relation to other species of *Phytophthora*. Lengths of branches represent posterior probability based on Maximum-likelihood analysis of the dataset. Sequences generated in this study are in boldface with GenBank accession identifiers following the isolate number.


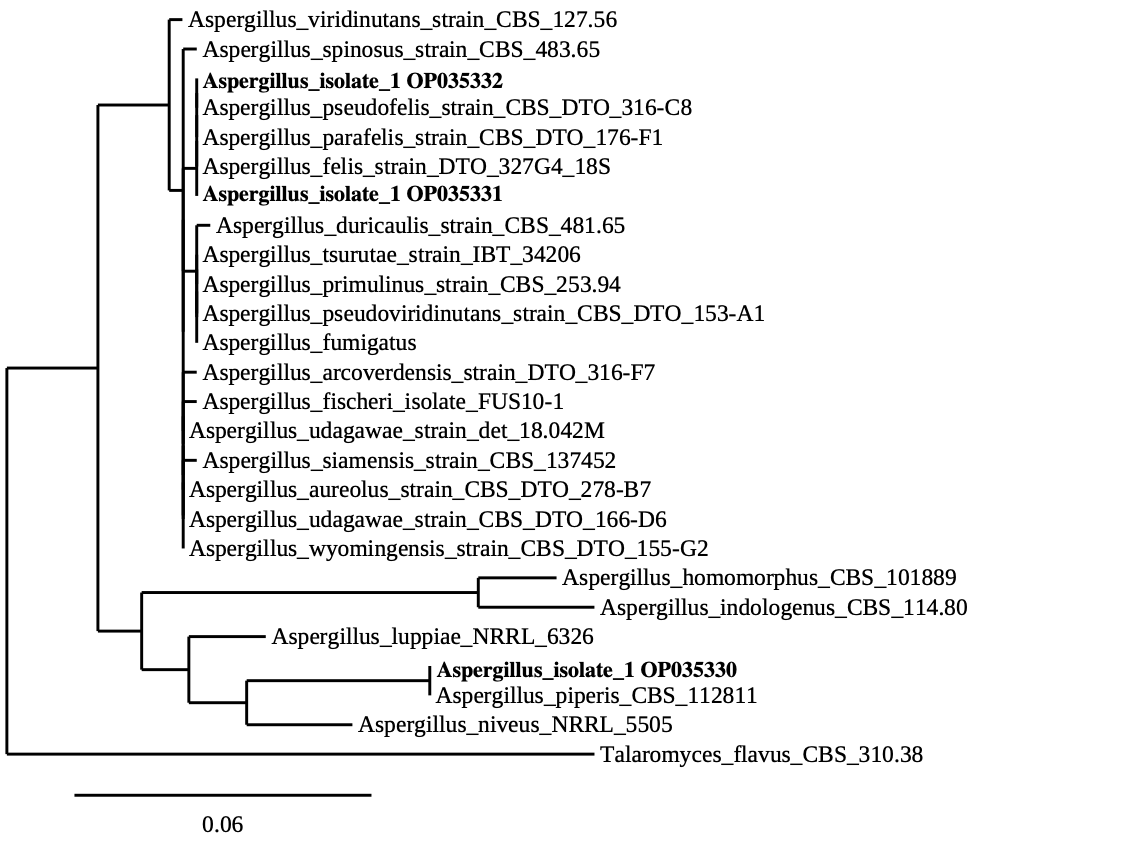


**Figure S9** Tree based on ITS sequences showing the phylogenetic position of isolates obtained in this study in relation to other species of *Aspergillus*. Lengths of branches represent posterior probability based on Maximum-likelihood analysis of the dataset. Sequences generated in this study are in boldface with GenBank accession identifiers following the isolate number.


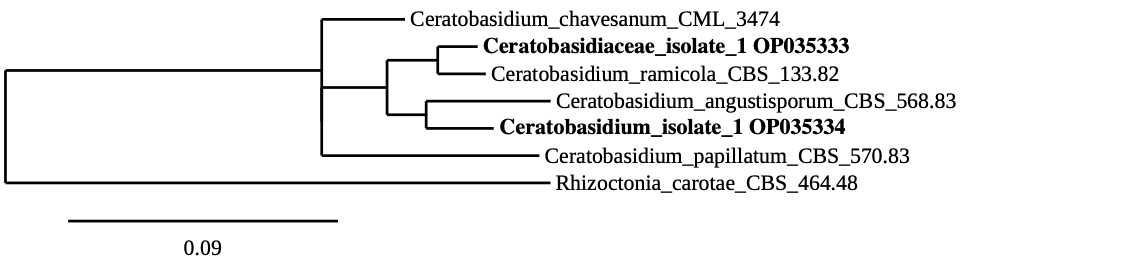


**Figure S10** Tree based on ITS sequences showing the phylogenetic position of isolates obtained in this study in relation to other species in the Ceratobasidiaceae. Lengths of branches represent posterior probability based on Maximum-likelihood analysis of the dataset. Sequences generated in this study are in boldface with GenBank accession identifiers following the isolate number.


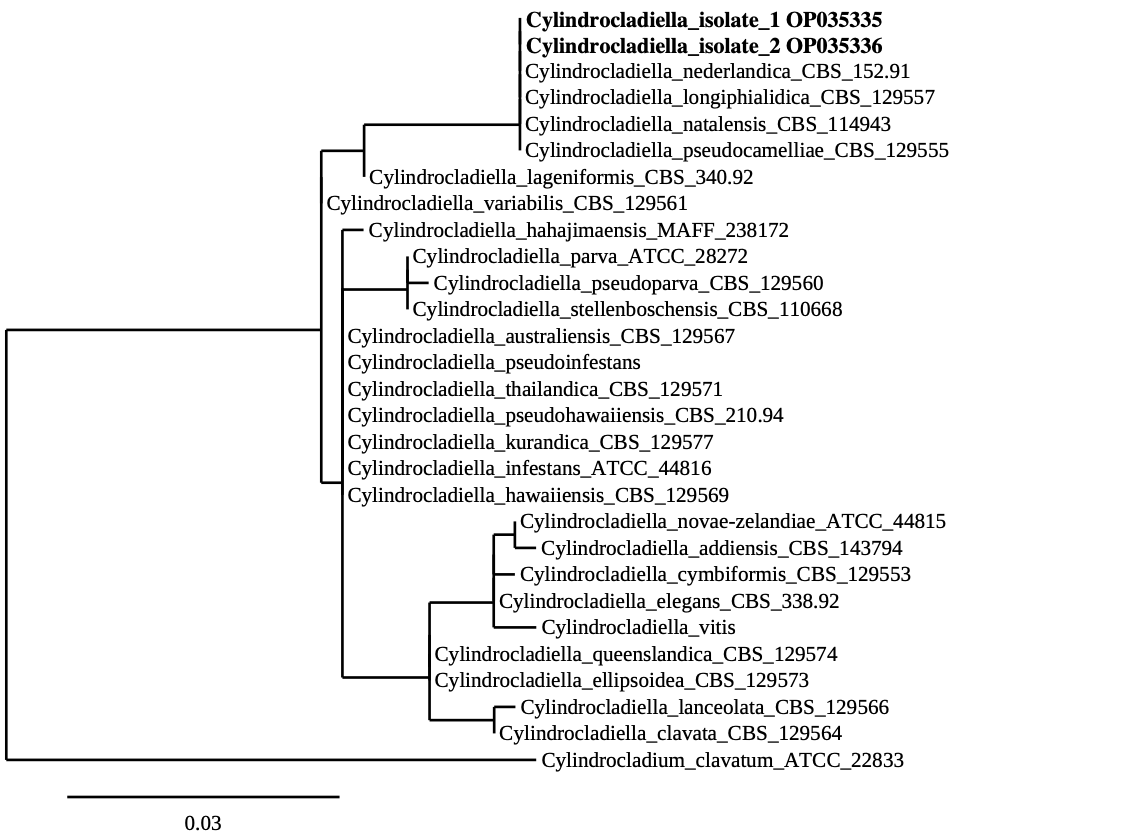


**Figure S11** Tree based on ITS sequences showing the phylogenetic position of isolates obtained in this study in relation to other species of *Cylindricocladiella*. Lengths of branches represent posterior probability based on Maximum-likelihood analysis of the dataset. Sequences generated in this study are in boldface with GenBank accession identifiers following the isolate number.

**
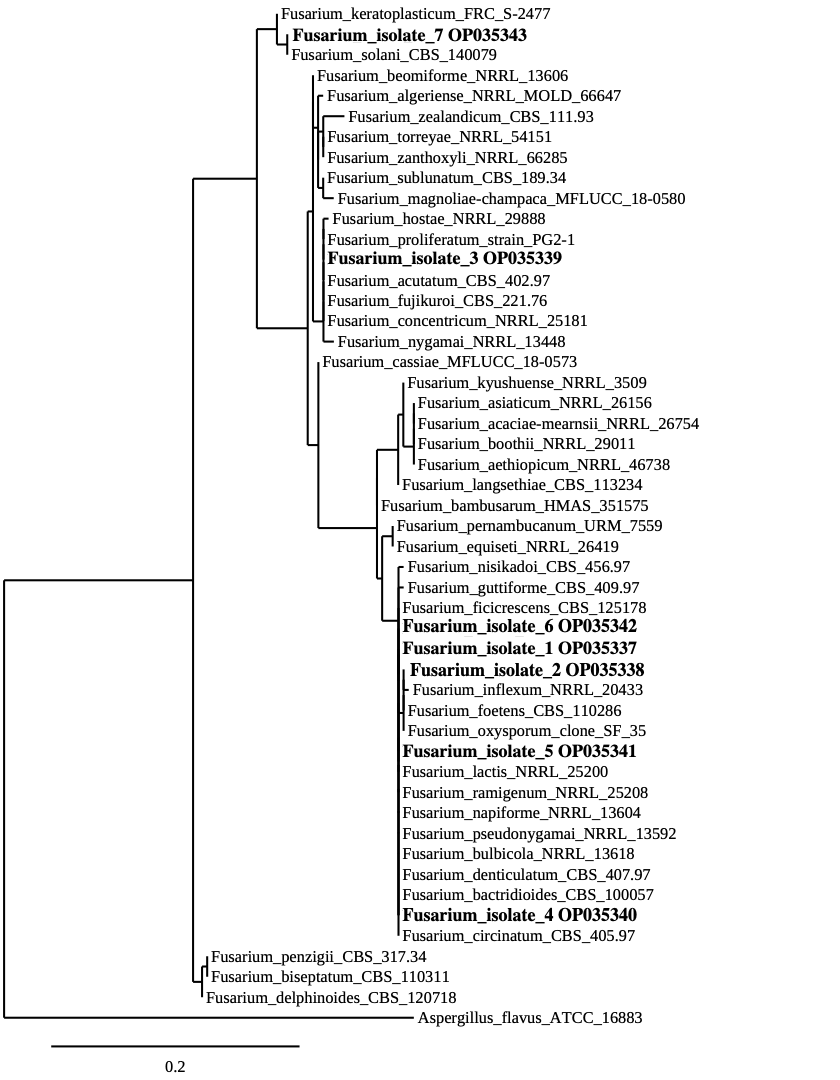
**

**Figure S12** Tree based on ITS sequences showing the phylogenetic position of isolates obtained in this study in relation to other species of *Fusarium*. Lengths of branches represent posterior probability based on Maximum-likelihood analysis of the dataset. Sequences generated in this study are in boldface with GenBank accession identifiers following the isolate number.


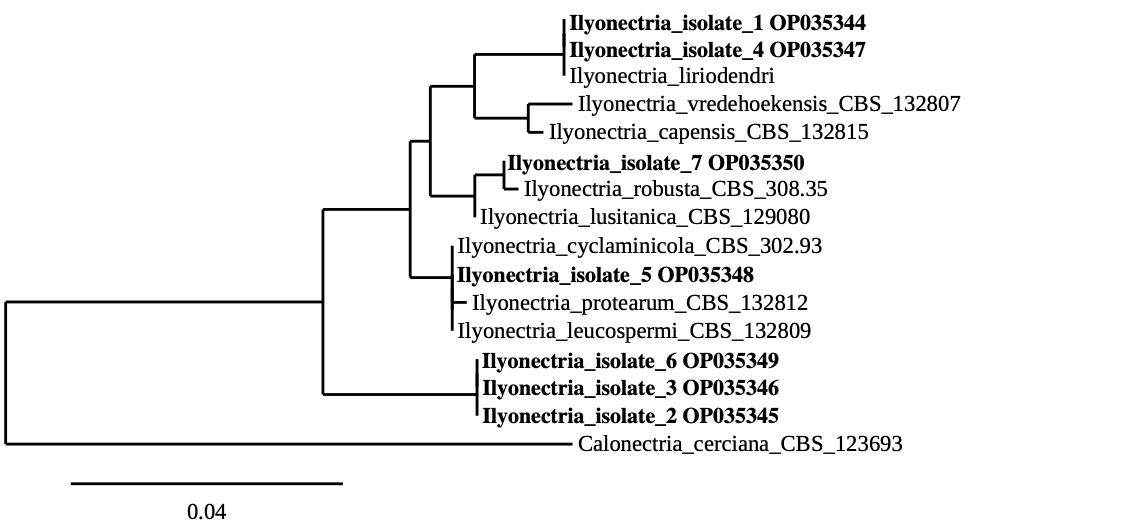


**Figure S13** Tree based on ITS sequences showing the phylogenetic position of isolates obtained in this study in relation to other species of *Ilyonectria*. Lengths of branches represent posterior probability based on Maximum-likelihood analysis of the dataset. Sequences generated in this study are in boldface with GenBank accession identifiers following the isolate number.


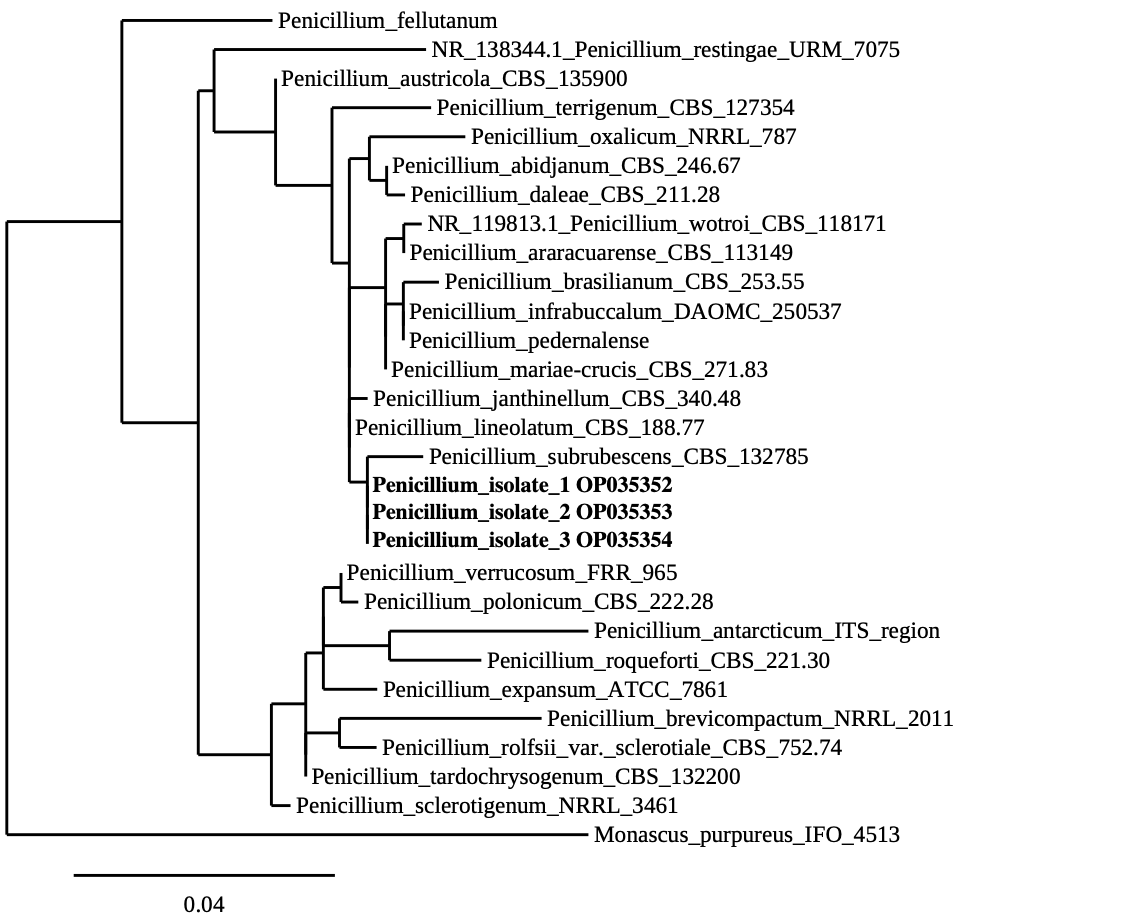


**Figure S14** Tree based on ITS sequences showing the phylogenetic position of isolates obtained in this study in relation to other species of *Penicillium*. Lengths of branches represent posterior probability based on Maximum-likelihood analysis of the dataset. Sequences generated in this study are in boldface with GenBank accession identifiers following the isolate number.


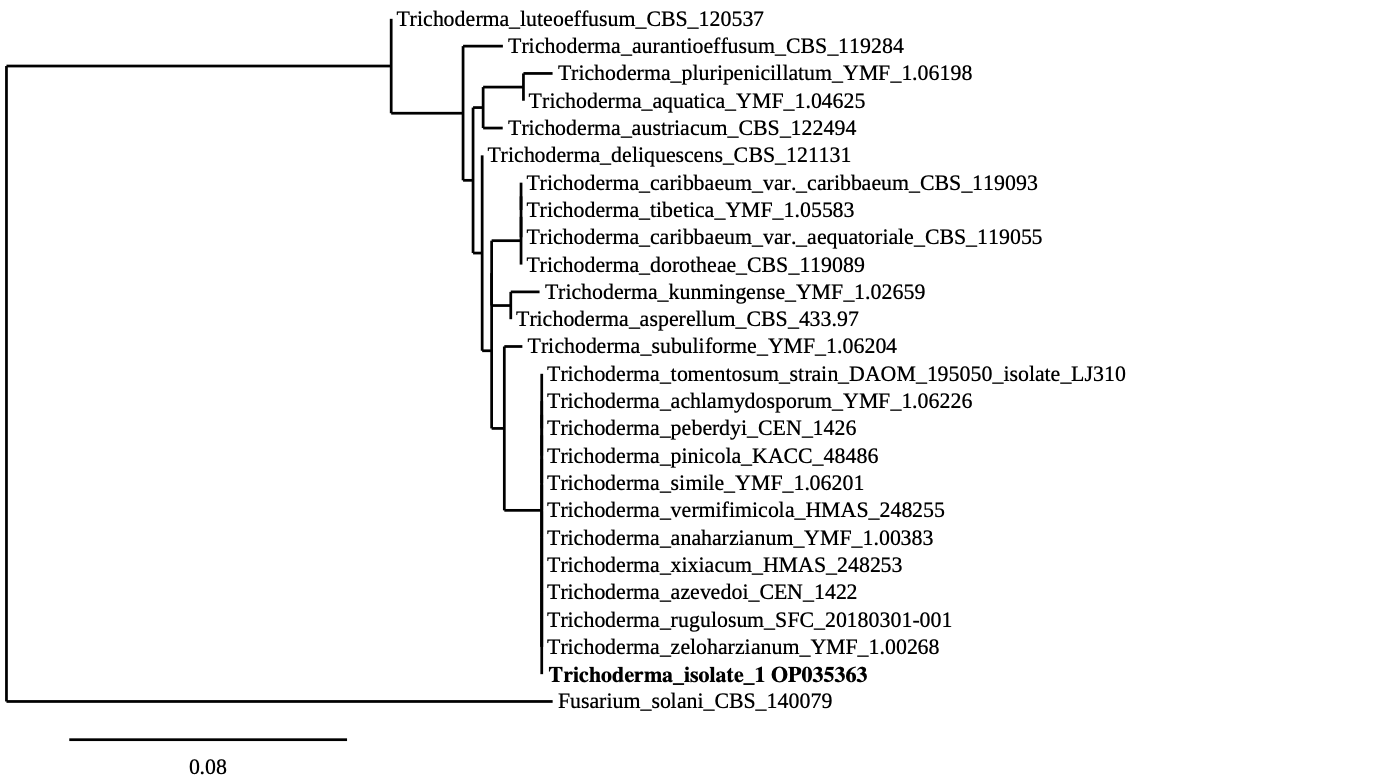


**Figure S15** Tree based on ITS sequences showing the phylogenetic position of isolates obtained in this study in relation to other species of *Trichoderma*. Lengths of branches represent posterior probability based on Maximum-likelihood analysis of the dataset. Sequences generated in this study are in boldface with GenBank accession identifiers following the isolate number.
